# Crafting the architecture of biomass-derived activated carbon *via* electrochemical insights for supercapacitors: a review

**DOI:** 10.1039/d4ra07682f

**Published:** 2025-01-24

**Authors:** T. Manimekala, R. Sivasubramanian, Mushtaq Ahmad Dar, Gnanaprakash Dharmalingam

**Affiliations:** a Electrochemical Sensors and Energy Materials Laboratory, Department of Nanoscience and Technology, PSG Institute of Advanced Studies Peelamedu Coimbatore-641 004 Tamilnadu India; b Department of Chemistry, Amrita School of Physical Sciences, Amrita Vishwa Vidyapeetham Amaravati Andhra Pradesh India; c Center of Excellence for Research in Engineering Materials, Deanship of Scientific Research (DSR), King Saud University Riyadh 11421 Saudi Arabia; d Plasmonic Nanomaterials Laboratory, Department of Nanoscience and Technology, PSG Institute of Advanced Studies Peelamedu Coimbatore-641 004 Tamilnadu India dgp@psgias.ac.in

## Abstract

Escalating energy demands have often ignited ground-breaking innovations in the current era of electrochemical energy storage systems. Supercapacitors (SCs) have emerged as frontrunners in this regard owing to their exclusive features such ultra-high cyclic stability, power density, and ability to be derived from sustainable sources. Despite their promising attributes, they typically fail in terms of energy density, which poses a significant hindrance to their widespread commercialization. Hence, researchers have been exploring different cutting-edge technologies to address these challenges. This review focuses on biomass-derived activated carbon (BDAC) as a promising material for SCs. Initially, the methodology and key factors involved in synthesising BDAC, including crafting the building blocks of SCs, is detailed. Further, various conventional and novel material characterization techniques are examined, highlighting important insights from different biomass sources. This comprehensive investigation seeks to deepen our understanding of the properties of materials and their significance in various applications. Next, the architectural concepts of SCs, including their construction and energy storage mechanisms, are highlighted. Finally, the translation of the unravelled BDAC metrics into promising SCs is reviewed with comprehensive device-level visualisations and quantifications of the electrochemical performance of SCs using various techniques, including cyclic voltammetry (CV), galvanostatic charge–discharge test (GCD), electrochemical impedance spectroscopy (EIS), cyclic tests (CT), voltage holding tests (VHT) and self-discharge tests (SDT). The review is concluded with a discussion that overviews peanut-shell-derived activated carbon as it is a common and promising source in our geographical setting. Overall, the review explores the current and futuristic pivotal roles of BDAC in the broad field of energy storage, especially in SC construction and commercialisation.

## Abstract 2

Escalating energy demands have often ignited ground-breaking innovations in the current era of electrochemical energy storage systems.

## Graphical abstract

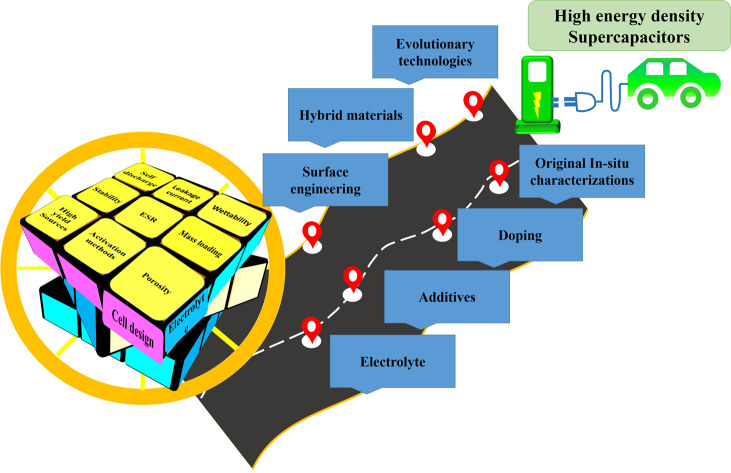

## 1 Introduction

The depletion of fossil fuels and environmental pollution propel the development of efficient and environment-friendly green energy storage devices.^1,2^ In this realm, supercapacitors (SCs) are efficient electrochemical storage devices that can store electrical energy *via* electrostatic interactions, with high power densities (*P*_d_) (>10 kW kg^−1^), long cycle lives, rapid carbon charging/discharging rates, good reversibility, and wide operating temperatures (−40 °C to 70 °C).^3–6^ Thus, they are apt for applications with high power requirements, such as hybrid electric vehicles, portable electronic equipment, energy and power sectors, military and defence, aerospace, and aviation.^7–9^ Worldwide, as the demand for SCs is expected to rise rapidly at a CAGR of 20.17% during 2023–2032, reaching a market value of US $ 27.7 billion by 2032, a myriad of companies have invested in propelling SC research and commercialisation.^10^ Conventional capacitors have mF- or μF-scale capacitance, which depends largely on the area and distance between the electrodes.^11^ SCs comprise two electrodes sandwiched between electrolyte-soaked ion separators. Electrolytes that contain solvated ions and a thin, ion-permeable, microporous membrane separator permit the free passage of ions between them.^12^ This leads to higher *P*_d_ than those of batteries, and batteries involve slower electrochemical reactions. Hence, the energy density (*E*_d_) of SCs is relatively higher than those of conventional capacitors, but their *P*_d_ is vastly superior to those of batteries.^13,14^ Thus, they serve in part to bridge the gap between rechargeable batteries and conventional capacitors.^15,16^

The electrochemical performance of SCs relies on the type of electrode material (*i.e.*, carbon/metal oxide/polymer-based)^17–19^ and their mass loading,^20^ type (*i.e.* aqueous, non-aqueous, or solid-state)^21–23^ and molar concentration of the electrolyte,^24,25^ operating potential window,^26^ binders,^27^ current collectors (*i.e.* foil, foam, cloth, or sheet),^28–30^ type of separators,^31,32^ cell type (symmetric or asymmetric),^33,34^ cell assembly type (coin cell, cylindrical cell, prismatic cell or pouch cell),^35,36^*etc.* However, SCs are limited by their low *E*_d_, which is dictated by their specific capacitance (*C*_s_). Current SC research focuses on multiple aspects, such as the design and fabrication of separators,^31^ development of electrolytes,^37^ examining the ageing properties of SC cell components *via in situ* and *ex situ* techniques^38^ and developing electrode materials from widely available sources, such as biomass, to enhance the *E*_d_.

The emergence of flexible and portable electronic devices has created a high demand for electrochemical energy storage systems with exceptional energy density and power density. A primary obstacle in this domain is the development of flexible electrodes capable of meeting the energy density requirement. A promising solution to this challenge involves fabricating 3-D interconnected hybrid nanostructures that can serve as flexible current collectors. This structure offers a high surface-to-volume ratio, enabling high mass loadings of electroactive materials while maintaining excellent ohmic contact between the electrodes and current collectors.^34^ Other key challenges are in selecting novel electrode materials in the nanoscale, designing electrodes, understanding the fundamental differences between electrochemical capacitors and battery-type materials, and developing basic techniques to analyse capacitive and pseudocapacitive behaviours.^39^

Carbon has different allotropes, such as graphite, diamond in the bulk phase and fullerene, carbon nanotubes (CNT), and graphene, in the nanoscale. Among carbonaceous materials, Biomass-Derived Activated Carbon (BDAC) is deemed to be crucial for commercial applications primarily due to its easy availability and low-cost production with a high yield compared with other carbon sources, such as carbide-derived carbon,^40^ graphene,^41^ and metal–organic frameworks.^42^ BDAC from various biomass sources have been extensively investigated for sustainable energy storage because of their meritorious physicochemical properties, such as high surface area, tunable porosity, chemical stability, good electrical conductivity, environment friendliness, and outstanding cycling stability.^43^

This review aims to provide an overview of the research on BDAC-based SCs. Due to the prevalence of biomass and the focus on SCs based on carbonaceous materials, here, we have reviewed different viewpoints and aspects of SCs based on carbonaceous materials, such as the significance of biomass, different sources, tuning material properties, BDAC-relevant SC configurations and understanding the relationship between BDAC and the electrochemical performance using powerful instrumentational techniques. We also comprehensively discuss peanut shells as a source of biomass-derived carbon that is prevalent in our geography and surmise the exclusive benefits of peanut shell-derived activated carbon (PSAC).

Contextually, this review is divided into three broad sections. The first section is on BDAC, starting from the interest in carbon materials for SC applications and BDAC metrics relevant to SCs, such as surface area (m^2^ g^−1^), specific capacitance (*C*_s_) (F g^−1^), *E*_d_ (W h g^−1^), *P*_d_ (W g^−1^), capacitance retention (%) and post device cycling, reported so far. It further focuses on the synthesis, types, and activation mechanisms (Subsection 2), while BDAC characterization techniques are reviewed in Subsection 3. The second section of the review explains the applicability of BDAC in SCs (Subsection 4), including generic SC principles, SC construction, the working mechanisms, and specific characterization techniques for SCs that have been integrated with BDAC materials. The third and final broad section of the review concentrates on an essential subset of BDAC that is directly relevant to the geographical location of our group, namely the peanut shell-derived activated carbon (PSAC) (Subsection 5).

## 2 Carbon for SCs and carbon derived from nature: exploring BDAC

### 2.1 Architectures and morphologies of BDAC for optimal SC performance

In a SC, low tortuosity and a 3-dimensional (3D) hierarchical pore structure provide an optimal avenue for hydrated ions to move through the active material, resulting in improved performance, while uniform pore size distribution (PSD) offers consistent and shorter channels for the diffusion of the hydrated ions. The presence of performance-enhancing functionalities, such as functional groups that can be activated to enhance adsorption and charge mediation, is also a vital factor. For instance, an oxygen-containing functional group on the surface of the BDAC will enhance the hydrophilicity and the wettability of the electrode.^44,45^ Hence, it can be intuitively proposed that a biomass source with high carbon content (and consequently lower ash content), a 3D hierarchical porous structure and surface-enhancing functionalities are beneficial for SC applications.^46,47^

A 3D interconnected hierarchical porous activated carbon (HPAC) is a complex material characterised by pores of different sizes across multiple length scales, forming a three-dimensional interconnected framework. It usually contains various kinds of pores, such as micropores, mesopores, and micropores.^48^ Typically, 3D interconnected HPAC allows fast charge transfer, which acts as an avenue for the solvated ions in the electrolyte. One of the essential criteria of an electrode material is the availability of a large surface area that facilitates abundant electroactive surface sites.^49^ Hence, hierarchical pore formation offers higher capacitance than homogenous pores. Mainly, micropores (<2 nm) can store electric charges, mesopores (2–50 nm) provide diffusion pathways for ions, and macropores (>50 nm) act as reservoirs.^50^ Moreover, surface defects on BDAC provide dangling bonds that serve as electrochemically reactive sites for the solvated ions in the electrolyte.^51^ A thorough exploration of 3D interconnected HPAC and its importance by Yan *et al.* is considered a considerably important publication in the field of BDAC-derived SCs.^52^ For example, angstrom-sized HPAC has been derived from onions through a direct pyrolysis method. Its N_2_ adsorption–desorption showed a type-I isotherm, which indicates a large number of micropores. Moreover, a sharp peak at 0.7 nm indicated a very short micropore distribution.^48^ Nanoporous structures with a 3D interconnected hierarchical porous architecture were obtained from cow dung at varying activation temperatures, with a substantial surface area of 2457 m^2^ g^−1^. This enhanced porosity is attributed to the removal of the dangling bonds and volatile components from the aromatic structure, resulting in a combination of nanopores and mesopores.^53^ Although template-assisted methods are preferred for generating ordered porous carbon, their practical implementation is hampered by complicated processing steps, the use of substantial amounts of activating agents, and template dependence.

Yet, morphologies that differ from the proposed combinations have demonstrated high performance as well; for example, a sheet-like structure facilitates direct pathways for ions to diffuse through the electrode material, effectively shortening the diffusion pathway, which leads to faster ion kinetics. A balanced combination of high surface area and mesopore volume can also be achieved by carefully choosing processing conditions, such as pre-carbonization of wheat husk using a salt sealing technique in the air followed by KOH activation. The combination of molten KCl and NaCl salts played a dual role in the pre-carbonization step, acting both as a template to generate mesopores and a shielding agent that mitigated oxidation in the carbon network at high temperatures. Scanning Electron Microscopy (SEM) images indicated the presence of discontinuous graphite stripes formed owing to the strong etching of Cl^−^ ions in the molten salt mixture. The sample prepared at 700 °C had the largest surface area of 2721 m^2^ g^−1^ and a mesopore volume of 0.87 cm^3^ g^−1^, and this material exhibited a *C*_s_ of 346 F g^−1^ at 1.0 A g^−1^ and retained 98.59% of the initial capacitance after 30 000 cycles at 5.0 A g^−1^.^54^

### 2.2 Engineering biomass into activated carbon

**Fig. 1:**
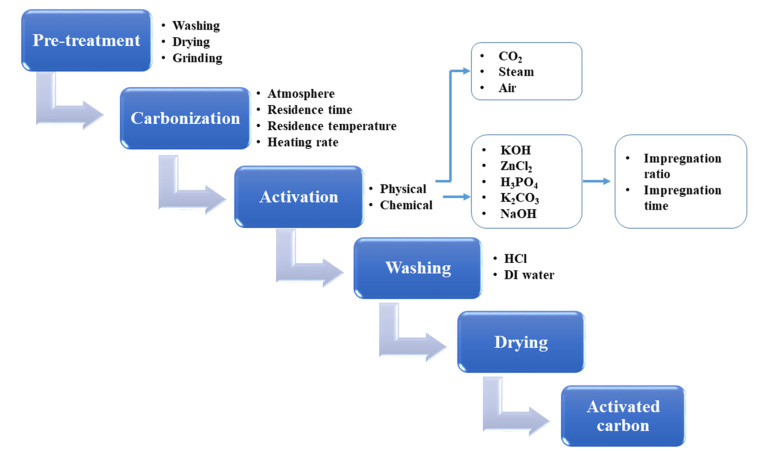
A detailed protocol on crafting high-performance BDACs.

BDAC is broadly synthesized by a two-step process involving carbonization and activation. Differing sub-processes are usually employed to manipulate parameters like the carbonization and activation temperature, heating rate, residence time, activating agents, impregnation ratio, impregnation time, and heteroatom dopant to different degrees. Fig. 1 represents the important processes and sub-process modifications involved in the synthesis of BDAC.

A single-step chemical activation combines the carbonization (pyrolysis) and activation steps. The biomass precursor is directly impregnated in the chemical activating agents *via* cleavage of the aryl-ether bonds and the release of volatile by-products that leave pores in the carbon skeleton.^55^ Higher activation temperatures may lead to high surface areas and pore volumes, but exceeding the limits has been reported to cause pore collapse. This is due to the decomposition of carbon at high temperatures and the rapid release of volatile molecules that had been a part of the pore structure. Another major cause of pore collapse is high heating rates; slower rates permit a more controlled release of volatile gases and can reduce pore collapse.^52^

BDAC synthesized *via* one-step activation typically has low packing density and yield (the latter is mitigated by ensuring proper deoxygenation during the activation protocols^56^). The electrode packing density can be calculated using the following eqn (1),1
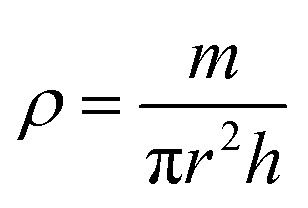
where *ρ* is the electrode packing density, *m* is the mass of the active electrode material, *h* is the thickness of the electrode and *r* is the radius of the electrode.^57^

In dual-step activation, carbonization followed by activation enhances the carbon content and decreases the ratio of oxygen to carbon. During the activation step, fewer oxidizing gases escape from the carbon structure, leading to the formation of a controlled porous network.^58,59^

### 2.3 Critical factors in BDAC synthesis

#### 2.3.1 Pre-treatment

Before the initial carbonization step, priming the biomass source can be beneficial in many ways. Hierarchical porous activated carbon (HPAC) from sugarcane bagasse prepared through hydrothermal-based and ethanol-soaking pre-treatments before chemical/physical activation showed increased microporosity, *C*_s_, and rate performance compared with other BDAC electrodes prepared without the pre-treatment step.^60^

Swelling-assisted synthesis for biomass cellulose is another strategy to produce HPAC. Using this pre-treatment, HPAC was produced from cotton by soaking it in NaOH/urea solution. This was followed by a carbonization process that resulted in swelling of the cellulose, which significantly destroyed its inter and intra-hydrogen molecular bonding, thus reducing the degree of polymerisation and increasing the surface area of the resultant HPAC. This is attributed to multiple reasons in studies that detail the mechanism of cellulose degradation, such as the ability of Na^+^ ions to infiltrate easily into cellulose owing to the low ionic radius of and high charge density, the tendency of the urea hydrates formed during the dissolution process to prevent cellulose chains from approaching each other and hence retaining their dispersed state, and the electron lone pairs that facilitate solvent ion exchange interactions.^61,62^ In biomass precursors with significant proportions of lignin in addition to cellulose, however, H_3_PO_4_ is the molecule of choice as it can cause effective degradation through the formation of pyrophosphates that are highly corrosive and oxidizing, thereby maximizing pore formation.^63^

#### 2.3.2 Activating agents

Of activation methods that involve physical and chemical processes, chemical activation has a few merits, such as shorter activation time, lower temperatures, more control over porosity, and a high char yield.^64^

Broadly, chemical activating agents can be categorized as alkaline (KOH, NaOH), acidic (H_2_SO_4_, H_3_PO_4_), oxidizing (H_2_O_2_, KMnO_4_, K_2_FeO_4_) and salts (K_2_CO_3_, FeCl_3_, FeCl_2_, KHCO_3_, ZnCl_2_).^63^ KOH is a suitable activating agent since it is eco-friendly relative to other activating agents and can also develop good porosity along with a narrow pore size distribution. The possible functional groups found on the surface of BDAC post-KOH activation are aromatic rings, primary alcohols, phenols, aldehydes, and carboxylic acids. The presence of surface functional heteroatomic groups, such as nitrogen (N_2_), oxygen (O_2_), and sulfur (S), can enhance the surface hydrophilicity, especially in aqueous electrolytes, thereby promoting charge transfer at the electrode/electrolyte interface. The KOH-to-biomass ratio is also a key factor in deciding the PSD.^65^

The activation process of BDAC using KOH is preferred as the activating agent is similar to an electrolyte. Therefore, the pore size of the electrode material becomes comparable to the ionic radius of the electrolyte ions, facilitating enhanced charge storage and transfer processes.^65,66^ Additionally, KOH removes lignin and hemicellulose components partially from the base biomass, intercalates into the carbon lattice, and acts as an electron donor, enhancing the gasification reaction (as shown in eqn (2)–(8)) that is responsible for the generation of pores within the carbon matrix.^67^ In these reactions, the consumption of carbon by O_2_ is catalyzed by the alkali metal, producing carbon monoxide and carbon dioxide. The detailed mechanism of pore formation during KOH activation is yet to be unravelled. Based on insights from published research, the following reactions elucidate the possible pathways of KOH activation.^68,69^ To begin with, at 400 °C, KOH dehydrates to K_2_O. Then water interacts with the carbon to produce CO, which further reacts with water and produces hydrogen (H_2_) and CO_2_. A reaction between K_2_O and CO_2_ takes place to form K_2_CO_3_.^70,71^

2 2KOH → K_2_O + H_2_O

3 C + H_2_O → CO + H_2_

4 CO + H_2_O → CO_2_ + H_2_

5 CO_2_ + K_2_O → K_2_CO_3_

A secondary reaction also happens to produce metallic K and K_2_CO_3._

6 6KOH + C → 2K + 3H_2_ + 2K_2_CO_3_

As temperature increases to 700 °C, K_2_CO_3_ is decomposed to K_2_O and CO_2_ which is further reduced to CO.

7 K_2_CO_3_ → K_2_O + CO_2_

8 CO_2_ + C → 2CO

The gaseous by-products of KOH decomposition, such as H_2_O, CO, and CO_2_, formed during the etching of the carbon matrix create varying degrees of porosity on and within the BDAC surface. Hence, two processes happen during the production of activated carbon *via* KOH activation. The reduction of hydroxides to produce free metallic potassium ions (K^+^), their penetration into the carbon lattice to form intercalated K^+^, and the eventual removal of K^+^ from the carbon matrix results in the creation and expansion of the pore network, improving the surface area and porosity. During KOH decomposition, O_2_ and O_2_-containing groups (such as H_2_O, CO, and CO_2_) chemically etch the carbon, introducing detrimental defects and disorder in the carbon structure. The existing covalent bonds are broken by the O_2_-containing compounds, polymerized monomers, and dangling bonds. Notably, while the thrusting process of electrolyte decomposition is facilitated by unpaired electrons, parasitic faradaic reactions also occur at the oxidized carbon sites, which can considerably influence the final morphology.^72^

The degree of activation depends on the KOH : BDAC weight ratio and the activation temperature,^73^ of which the latter plays a role in pore formation and increasing the specific surface area. However, pore formation and the increase in specific surface area do not scale with the temperature, and in fact, detrimentally high activation temperatures lead to pore shrinkage and lower yield^74^ due to increased release of the as-formed volatile aromatic molecules that readily escape from the carbonized char.^75^ The pore size of a BDAC material must be comparable to the size of the solvated electrolyte ions, along with a high specific surface area and narrow size distribution, which can be achieved by using BDAC precursors that have high cellulose and low lignin content.^59^ Micropores often lead to a significant fall in *P*_d_ as they elevate the equivalent series resistance (ESR), particularly in organic and ionic liquid electrolytes that offer high working voltage windows and hence can enhance charge migration better than those with lower working voltages. Furthermore, the electrolyte species can easily be accommodated in the micropore volume, limiting the operating potential and *E*_d_. Mesopores can store more ions than micropores, as the ESR brought on by the desolvation process may cancel out, resulting in good rate capability. Nevertheless, excessively large mesopores can reduce the specific surface area of the BDAC, directly correlating with the electric double-layer capacitance (EDLC) performance. Hence, an effective way to boost reversible capacity and raise rate capability is by narrowing the PSD since it greatly reduces undesired ion scattering and improves the kinetics of the electrode reactions.^76^

CaCl_2_ is another chemical activating agent that has been used for the production of nitrogen-rich porous three-dimensional AC (3D-NPC) from sugarcane bagasse *via* a single-step activation in the presence of urea, which acts as an expanding agent and N_2_ source.^77^ In another work, cotton-stalk-based BDAC with an interconnected hierarchical porous structure with balanced mesopores and micropores was realised by a simultaneous activation strategy using KMnO_4_ and KOH. The intense interaction between KMnO_4_ and lignocellulose led to bond breakage at the ether aldehyde and hydroxyl groups to modify their architecture, resulting in two distinct pore size distributions, *viz.* 0.5–2 nm and 4–20 nm, with a mean pore width of 3.5 nm.^78^

ZnCl_2_ treatment has been proven effective for banana fibres, yielding a surface area of 1097 m^2^ g^−1^, which exceeds those of KOH-treated samples. As ZnCl_2_ has a melting point (275 °C) lower than that of KOH (360 °C), it may more easily penetrate the carbon matrix. The dehydrating property of ZnCl_2_ plays a crucial role in the activation process, promoting the formation of a more developed carbon structure. Due to the presence of an incomplete electron orbital, the Zn atom is positively charged and readily attracts the lone pair of electrons on the O_2_ atom of the hydroxyl group. The key to removing moisture lies in the interaction of ZnCl_2_ with the carbon matrix, which weakens the bonds between the H_2_ and O_2_ atoms within the hydroxyl groups, making it easier to remove water molecules from the biomass. The dehydrating effect not only removes water but also promotes the formation of a more aromatic carbon structure by eliminating O-containing functional groups, which causes reshuffling of the carbon atoms and remaining H_2_ atoms within the carbon matrix. This results in denser cyclic structures, with alternating single and double bonds between the carbon atoms.^79^ However, a significant drawback of using ZnCl_2_ is that it necessitates the elimination of metal chloride residues in the post-activation step. Moreover, the yield of ZnCl_2_-treated BDAC is low compared with KOH treatment because it burns off a large amount of carbon during the activation process.

#### 2.3.3 Effect of heteroatom doping

A powerful tool for enhancing electrode wettability, accessibility of the available surface area, and capacitance is doping heteroatoms, such as N, S, boron (B), and phosphorous (P). This can also facilitate the contribution to pseudocapacitance *via* faradaic reactions.^80,81^ The carbon atoms in the sp^2^ lattice of graphite can accommodate the heteroatoms.

Among heteroatoms, N is widely used as a doping agent in carbon matrixes because of its easy integration into the carbon structure and higher electronegativity than those of carbon and hydrogen atoms.^82^ Pyridinic and pyrrolic nitrogen from the resulting N-containing functional groups stimulate a positive charge on the adjacent carbon atoms, increasing the number of electroactive sites on the surface of the carbon material to adsorb more solvated ions and promote electrode wettability.^83^ N doping in a carbon skeleton will also lead to the formation of defects that further elevate the number of active sites.^84^ N-doped carbon can be synthesized by incorporating nitrogen-containing precursors, such as urea, melamine, polypyrrole, and polyaniline, through *in situ* doping. These N-rich compounds are directly activated by the carbonaceous material or through *ex situ* doping, that is, the N source is introduced after the activation process, *i.e.*, during the thermal process. While *in situ* doping offers a more direct approach, it often results in a lower concentration of oxygenated nitrogen functional groups due to the need for higher activation temperatures as thermodynamics favour endothermic molecule/phase/morphology-forming reactions in tandem with the reactions responsible for doping.

Single-step carbonization of nitrogen-doped honeycomb-like porous carbon (NPC-K) sourced from dumpling flour using KOH as an activating agent and urea as the N_2_ source led to a high surface area of 2856.3 m^2^ g^−1^ alongside a high *C*_s_ of 311 F g^−1^.^83^

Urea can serve as a low-cost nitrogen source and provide a more compact carbon structure than other sources. Cellulose-derived N-doped porous carbon was produced using urea at various molar ratios with respect to H_2_O. The surface area decreased from 552 to 450 m^2^ g^−1^ with increasing urea concentration. Nitrogen and carbon elements in urea lead to pore shrinkage and decrease the surface area. However, N-doped porous carbon with 3.61% N content exhibited higher *C*_s_ (179 F g^−1^) than N_2_-free porous carbon (129 F g^−1^). This led to the conclusion that nitrogen functional groups significantly increase the *C*_s_ even with a lower surface area by providing a hydrophilic environment and increasing wettability.^85^

Three different N-containing molecules (NH_4_Cl, (NH_4_)_2_CO_3,_ and urea) were employed as porogens and dopants to prepare N-doped hierarchical porous carbon materials from camellia pollen. Since urea has the highest nitrogen content, it was theorized to produce the highest *C*_s_ of 300 F g^−1^ at 1 A g^−1^. This indicates that N-containing species and O_2_ species on the surface of the carbon framework contribute chiefly to pseudocapacitance response. Based on this, urea can be considered an excellent porogen and N_2_-dopant because it resulted in the best *E*_d_ to the tune of 14.3 W h kg^−1^ in the symmetrical configuration.^86^ For the preparation of orange peel-derived BDAC *via* KOH activation, melamine was incorporated as the nitrogen source. When N-doped carbon was mixed with KOH for further activation at an impregnation ratio of 1 : 2, the melamine-doped material achieved the highest surface area of 1577 m^2^ g^−1^.^87^

Theoretical models have been employed to delve deeper into N-doping mechanics in carbon-based SCs. A prediction of synergistic interactions of the anions (BF_4_^−^) and cations (TEA^+^) of an organic electrolyte (tetraethyl ammonium tetrafluoroborate) with various carbon surface constructions, including pristine carbon surface, pyrrolic N-modified carbon surface, graphitic N-modified carbon surface, and pyridine N-modified carbon surface, was confirmed based on the observation that N-doping alters the electron distribution within the carbon structure. It creates more electron-rich or electron-deficient regions near the doping sites, leading to an increase in the overall dipole moment. For anions, the adsorption energy increased from the pristine surface (−41.1 kJ mol^−1^) to the pyridinic N-modified surface (−66.6 kJ mol^−1^), and for cations, the adsorption energy increased from the pristine surface (−13.3 kJ mol^−1^) to the pyridinic N_2_-modified surface (−85.5 kJ mol^−1^). These findings demonstrate that doped N significantly interacts with the carbon surface and the electrolyte.^88^

*In situ* self-activation has recently emerged as an attractive method of producing porous biomass carbon. Without an extrinsic activating agent, cellulose-rich sources can be transformed directly into activated carbon, for example, by the etching effect of CO_2_, H_2,_ and H_2_O emissions when higher carbonization temperatures (>600 °C) are employed. N-doped micro-porous carbon from celery has thus been prepared *via* one-step *in situ* self-activation using this approach, and the etching effect facilitated the development of micropores, leading to a hierarchical porous structure.^89^

Similarly, O is also an attractive heteroatom dopant to improve hydrophilicity in BDAC with hydrophobic properties that impede interactions between the electrode material and the cations and anions of the electrolyte.^90^

#### 2.3.4 Impregnation time

The functional groups on the surface and pore structures can be altered by optimizing the impregnation time of the activating agent with the biomass or biochar. Miscanthus grass was activated in a single step using KOH by varying the impregnation time (6, 12, 18, 24, and 48 h). Impregnation for 18 h increased the micropore volume (from 0.215 to 0.262 cm^3^ g^−1^) while relatively decreasing the mesopore volume. Hemicellulose is rich in oxygen content (because of the hydroxyl group (–OH) attached to the backbone), while lignin is rich in carbon content (due to the aromatic ring structure); the acetyl and carbonyl groups (C=O) of hemicellulose and lignin were removed, while the C–O groups in the cellulose component increased, as evidenced by the increasing hydroxyl (–OH) and ester (C–O–C) group concentrations. This led to the highest structural defect (*I*_D_/*I*_G_ = 0.98) proportion compared with other impregnation durations, reducing the crystallinity of cellulose and promoting the hierarchical porous network. Notably, excessive impregnation durations (48 h) in the activating agent resulted in a decrease in micropore volume due to pore collapse, which detrimentally influenced the capacitive behaviour despite a minor increase in mesopore volume (10–40 nm).^91^ Crucially, this was attributed to the loss of interconnectivity between the pores after pore collapse, which reverses the expected enhancement in capacitive behaviour due to an increase in mesopore volume. This proves that the impregnation time can be tuned to tailor the structural and surface properties of BDAC and, importantly, pore interconnectivity.

The following section discusses the characterization of synthesized BDAC, after which a subset of BDAC, namely PSAC for SC applications, has been reviewed.

### 2.4 Impact of key characteristic on BDAC: a path to better SC

Key factors that contribute to the enhancement of the SC behaviour of BDAC are: the selection of biomass precursors rich in carbon content and low in ash content to enhance conductivity; careful selection of chemical (KOH, ZnCl_2_) or physical (CO_2_, steam) activating agents to achieve a high density of active sites for ion adsorption; careful control of the activation temperature and residence time to achieve a high specific surface area and favorable porous structure, for example, a 3D interconnected structure with a balanced proportion of meso, micro and macropores that can facilitate faster ion kinetics; modifying the surface with functional groups (in the carbon exoskeleton) and heteroatoms in the lattice to generate redox active sites, enhance wettability and improve electrolyte penetration into the pores; introduction of conductive polymers or metal oxides into the carbon network for better enhancement of capacitance, energy density, and power density. Proper washing and drying are necessary to remove impurities and chemical residuals and ensure the stability of the activated carbon.

## 3 Unravelling the internal structure of BDAC

In the subsequent segments, we summarise the major characterisation tools and techniques that reveal the internal structure and performance metrics of BDAC, including but not limited to the morphology, porosity, and electrical metrics, as well as the lattice-level organization, which manifest significantly differently depending on the source of the BDAC.

### 3.1 Thermal analyses

**Fig. 2:**
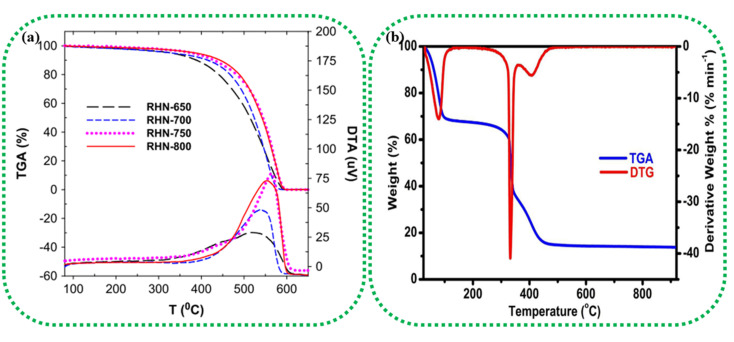
(a) TGA/DTA analysis of pinewood-derived activated carbon using different activating agents, such as H_3_PO_4_, KOH, and H_2_O_2_, and heat-only treatments. Reprinted with permission from ref. 93, copyright 2014, Elsevier. (b) TGA/DTG analysis of activated carbon prepared from rice husk at 850 °C under an O_2_ atmosphere. Reprinted with permission from ref. 94, copyright 2016, Elsevier.

Thermogravimetric analysis (TGA) and differential scanning calorimetry (DSC) can thoroughly analyse the thermal properties of the raw biomass sample and/or the BDAC.^92^ TGA helps identify the temperature ranges at which biomass components, such as cellulose, hemicellulose, and lignin, decompose. This helps in tuning the carbonization temperature and heating rate to achieve the required porosity levels and surface areas in the final BDAC. For example, rice husk-derived activated carbon materials prepared at different activating temperatures were characterized by TG-DTA in the temperature range of 80 to 650 °C, as illustrated in Fig. 2a. The carboxyl and lactonic groups present on the surface of the activated carbon led to the initial weight loss at around 350 °C. A more drastic weight loss was observed in the temperature range of 350 to 620 °C due to the oxidation of carbon, as represented by the exothermic peak at 550 °C in the DTA curves. After 620 °C, no weight loss was observed, and the cumulative weight loss in all samples was more significant than 93%.^93^ The TGA-DTG data of rice husk-derived activated carbon tested in an O_2_ environment at a heating rate of 10 °C min^−1^ are displayed in Fig. 2b. The curves show that 45% of the sample was lost in the temperature range of 30–166 °C due to moisture evaporation, and release of organic volatile components, and a second weight loss at 166–358 °C was due to the removal of cellulose and hemicellulose. Between 357 °C and 920 °C, the final weight loss (37.60%) occurred due to the decomposition of the lignin component.^94^

An okra powder sample exhibited three stages of weight loss due to the evaporation of water content, thermal decomposition of lignocellulose, and combustion in the final stage, at which the TGA curve stabilized because of the gradual decomposition of the residual sample into ash and carbon. From these results, the pyrolysis temperature was determined to be 600 °C. Two endothermic DSC peaks were observed at 319.8 °C and 542.8 °C, revealing the decomposition of its common functional groups, including carboxylic acids, alcohols, and esters.^95^

### 3.2 X-ray diffraction (XRD)

XRD gives information about the amorphous/crystalline structure, graphitic composition, lattice strains, modifications, *etc.*^96^ Typically, a broad peak at 23° corresponds to the (002) plane and indicates amorphous nature with a small crystalline region, and a narrower peak at 43° corresponds to both the (100) and (101) planes (typically denoted as (10) or (10*l*)), indicating graphitic nature.^97^ Diminished intensity and broadening of the peaks, therefore, indicate a decrease in the degree of graphitization of BDAC^49^ or higher content of amorphous carbon.

Generally, strain can originate from microstrain due to grain size effects and defects, such as vacancies, interstitials, dopants, and dislocations. BDAC is largely amorphous and turbostratic, so strain measurement is challenging. However, techniques like the Williamson–Hall method provide a semi-quantitative measure of strain from XRD data.^98,99^ High defect density in the carbon matrix has been shown to provide abundant active sites for improved EDLC behaviour.^100^

More often than not, the turbostratic (disorganized) BDAC carbon structure consists of sheets of carbon atoms (similar to graphite) stacked randomly and in a periodic fashion. The Braggs law and the Scherer equation hence become relevant, as in eqn (9). An inter-layer spacing (*d*_002_) describes the distance between the aromatic layers and *N* their number as in eqn (10).^101^ The total height of the stacked aromatic layer (the aromatic layer stacking height (*L*_c_)) is given by eqn (11), and the average crystallite size (*L*_a_) and number of layers is calculated using eqn (12) & (13).^102^ The peaks corresponding to the presence of just the single element are usually found at typical locations. One at 23°, which corresponds to a set of (002) planes, is used to calculate *L*_c_ and *d*_002_, and that at 43°, which corresponds to (10), can be used to deduce *L*_a_ using eqn (9).^103–106^

9 *nλ* = 2*d*_002_ sin(*θ*_002_)

10
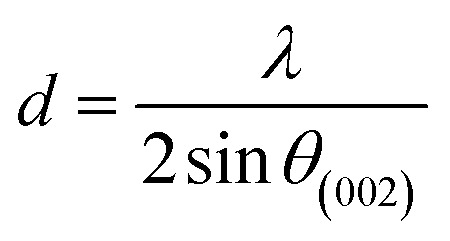
11
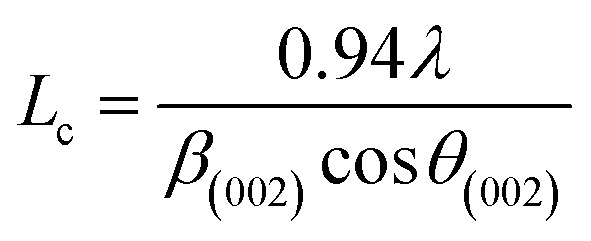
12
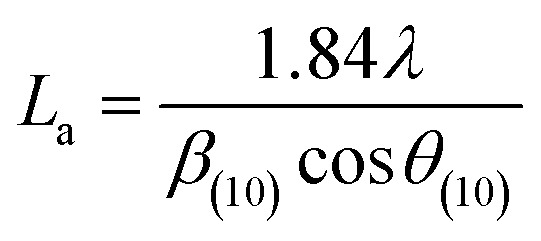
13
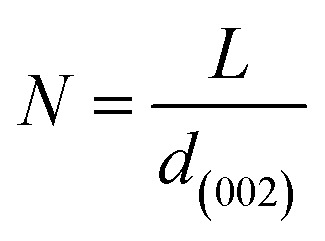
where *λ* is the wavelength of the XRD, *d*_(002)_ is the full width at half maximum of the (002) peak, and *θ* is the Bragg peak position. The measurement of *d*_(002)_ indicates the degree of periodicity in the stacking structure, whereas the (002) plane shows the distance between the aromatic ring layers. Obviously, as the content of volatile matter diminishes, *L*_c_ increases while the lattice spacing *d*_(002)_ decreases.^107^

**Fig. 3:**
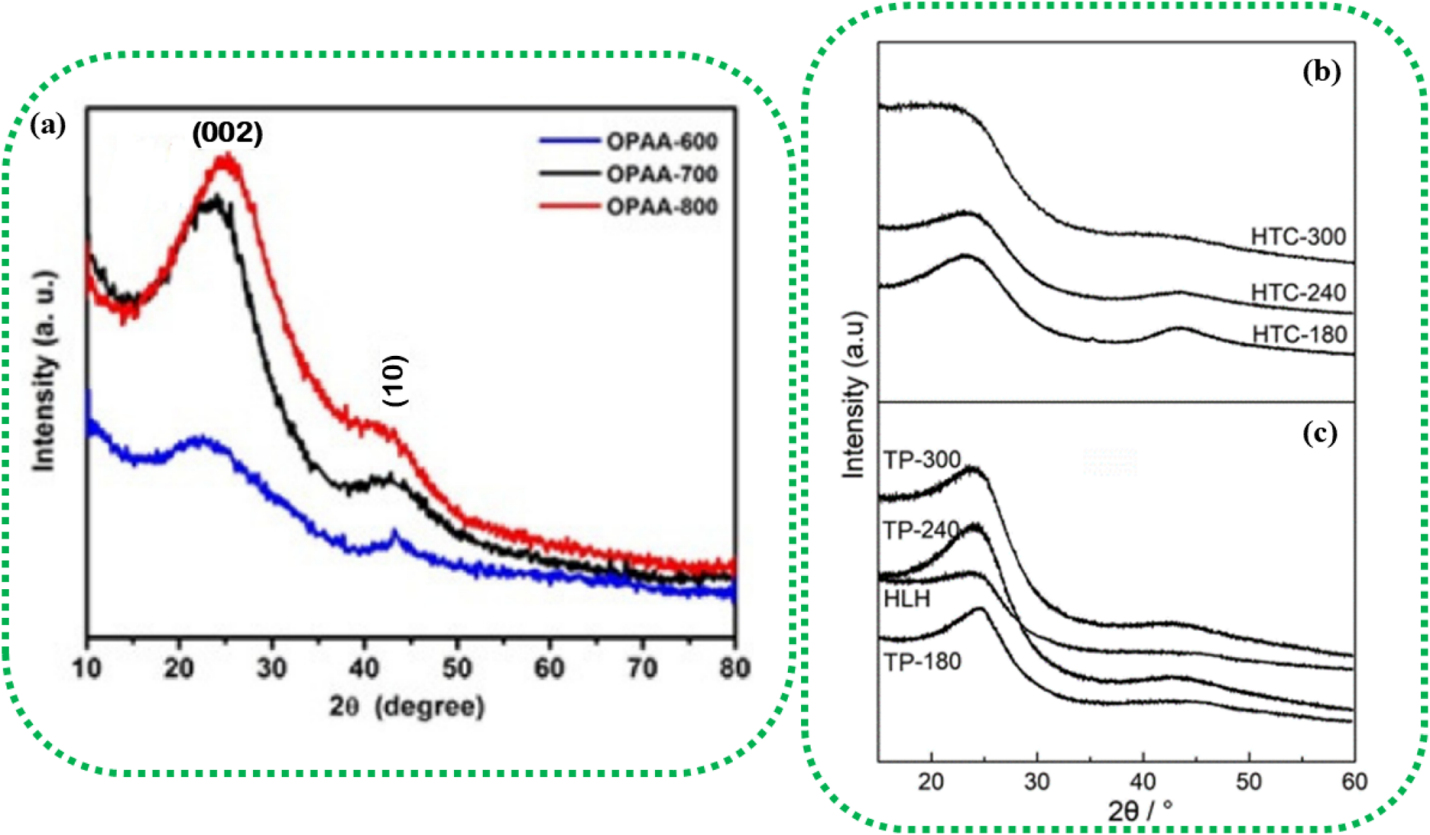
(a) XRD patterns of OPAC, demonstrating the influence of activation temperatures ranging from 600 °C to 800 °C on peak intensities. Reprinted with permission from ref. 108, copyright 2017, John Wiley and Sons. XRD profiles of (b) hydrochar produced by hydrothermal carbonization (HTC) at varying temperatures (180 °C, 240 °C, and 300 °C) (c) pyrochar produced by the traditional pyrolysis (TP) method at varying temperatures (180 °C, 240 °C, and 300 °C). Reprinted with permission from ref. 109 under a CC-BY-NC-ND.

Fig. 3a represents the XRD analysis of orange peel-derived activated carbon (OPAC) subjected to different activation temperatures. As the activation temperature increases from 600 °C to 800 °C, the (002) plane shows a slight shift toward the higher diffraction angles, denoting an increase in the graphitic properties.^108^ This increase in graphitic nature enhances the electrical conductivity of the BDAC materials, an important inference for designing BDAC synthesis protocols.^53^

The (002) and (10) planes linearly weaken with increasing hydrothermal temperature, and this is due to the formation of an aliphatic structure due to the breaking of the polymer chains in the crystalline structure, as seen in Fig. 3b, thus giving compositional insights regarding the BDAC. As an example, the weak diffraction of the asymmetrical peak at (002) for HLH (Huolinhe lignite) is shown in Fig. 3c, which denotes a high proportion of aliphatic structures. In contradiction to this diffraction weakening phenomenon, the peaks of the (002) and (10) planes of pyrochar-derived BDAC linearly strengthen with increasing temperature in Fig. 3c. This can be correlated to polymerization of the oxygen-containing groups by cross-linkage, which offsets the accompanying breakage of the aliphatic structure and creation of free-radical fragments. This aromatization reaction significantly promotes the formation of aromatic units found in pyrochar.^102,109^

### 3.3 Fourier transform infrared spectroscopy (FTIR)

FTIR is indispensable for SC research because of its significant merits of being non-destructive, sensitive, quantitative, and cost-effective, which are favorable to elucidating the surface and bulk properties of BDAC.^110^ Common oxygen-containing functional groups present on BDAC include carboxylic acids (COOH), ketones (C=O), ethers (C–O–C), and lactones (cyclic esters), which enhance ion adsorption by wetting the electrode surface. Amines (NH_2_), pyridinic nitrogen, and amides (CONH_2_) are examples of N-containing functional groups that can contribute to pseudocapacitance behaviour.

Table 1 depicts the important functional groups in BDAC that are typically analysed using FTIR.

**Table 1:** Overview of the functional groups of BDAC

Source	Peak position (cm^−1^)	Modes of vibrations	Reference
Willow catkins	3420 (broad peak)	N–H symmetric stretching vibration or hydrogen-bonded hydroxyl	111
	2925 & 2854	Aliphatic C–H stretching vibration (*i.e.* –CH_3_ and –CH_2_ in the alkyl group)	
	1740	C=O stretching (carbonyl, quinone, ester and carboxyl group)	
	1604	C–H deformation vibration	
	1460 & 1385	C–N stretching vibration	
	1246 (broad peak)	N=H in-plane deformation or C=C stretching vibration	
	1162	C–O vibrations in phenols, ethers, or esters	
Rice husk	3400–3500	O–H stretching	94
	1570–1580	Aromatic C=C bonds	
	1380 (sharp peak)	Traces of nitrate ions	
	1096	C–O bonds from esters and ethers	
Orange peel	3700–3100	O–H stretching vibrations of cellulose, hemicellulose, lignin, pectin, and absorbed water	112
	3003	Aliphatic saturated C–H stretching vibrations	
	1722	C=O stretching vibration of carboxyl groups of lignin, cellulose, and hemicellulose	
	1600 (weak peak)	C=C stretching vibration of aromatic or benzene rings in lignin	
	1500–1000	C–O structure (carbonyl, hydroxyl, ester, phenol, ether and methoxy group, carboxylate ion, ash content)	
	1463–1398	Aliphatic and aromatic groups in the plane deformation vibrations of methoxy, methyl, and methylene groups	
	1300–1000	C–O stretching vibration of carboxylic acids and alcohols	
	700 and 900	C–H bending with various degrees of substitution	
Vetch	3200–3800	O–H stretching vibration of the hydroxyl group	113
Oil palm kernel shell	3418 (broad & strong)	O–H stretching	114
	2924 & 2848	Asymmetric & symmetric C–H stretching	
	1748	Stretching of C=O	
	1580	Stretching mode of C=C	
	1386	Deformation mode of the C–H group	
	1070	C–O–C stretching mode	

### 3.4 Raman analysis

Raman spectroscopy can investigate the molecular structure, crystallinity, textural defects, and graphitization degree of BDAC.^115^ For BDAC, four characteristic bands in the region of 1800–1000 cm^−1^ are usually observed. The ratio between the D-band intensity to the G-band intensity (*R* = *I*_D_/*I*_G_) denotes the graphitization degree, and the crystallite size (*L*_a_) is extracted from the reciprocal of the *R*-value using eqn (14).^116^ A lower *I*_D_/*I*_G_ indicates a higher graphitization degree and thereby good electrical conductivity, while a higher ratio of *I*_D_/*I*_G_ indicates more amorphisation and correspondingly lower conductivity.^65,117^14
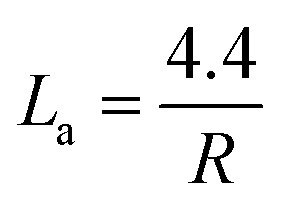


The D band represents the defect/disorder sites on the BDAC and appears between 1327 and 1360 cm^−1^, while the G band represents graphitic carbon and appears between 1572 and 1600 cm^−1^.^118–120^ The combination of D and G bands indicates multiple defects in the structure.^121,122^ Apart from these bands, Raman studies can reveal other BDAC signatures, including an I peak that originates from ionic impurities and defects (caused by heteroatom doping or heteroatom adsorption, for example) and a D′ peak arising from in-plane or stacking faults.^100^

**Fig. 4:**
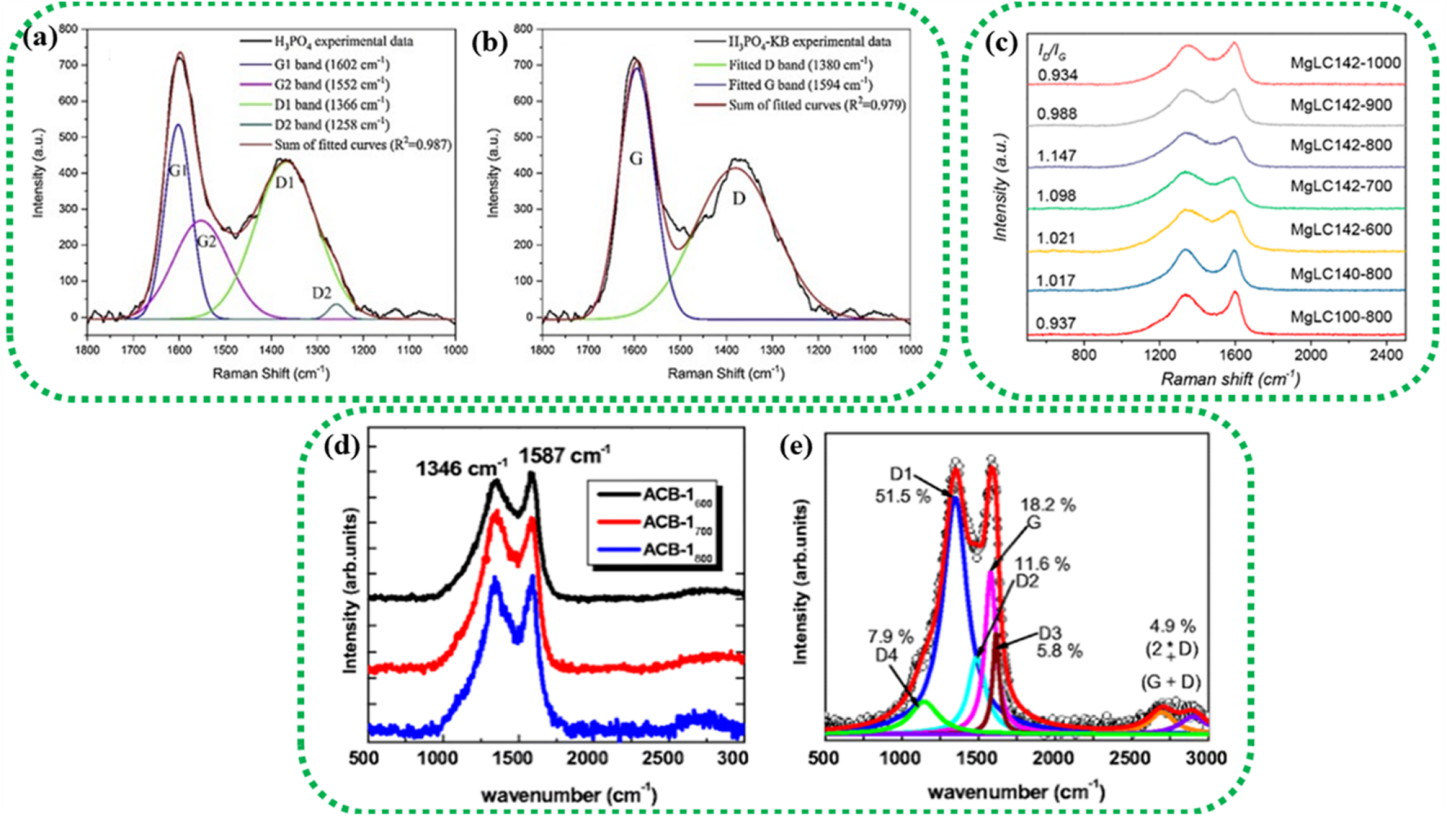
Raman spectra of different BDAC derived from (a and b) switchgrass; reprinted with permission from ref. 123, copyright 2020, Elsevier, (c) lotus root; reprinted with permission from ref. 124 copyright (2020), the American Chemical Society, and (d and e) tree bark; reprinted with permission from ref. 125, copyright (2017), Springer.

Representative spectra are displayed in Fig. 4(a and b), which are the Raman spectra of switchgrass-derived BDAC with sharper G1 and D2 peaks (indicating winding short basal planes with ordered bond angles) and broader G2 and D2 peaks (arising from the amorphous sp^2^ cluster with a disordered bond angle).^123^Fig. 4c shows the Raman spectrum of lotus-root-derived AC, comprising of D and G bands, which vary based on the different weight ratios of the lotus root powder, activating agent (MgCl_2_), and urea (N_2_-doping source), as well as pyrolysis temperature. An increase in the weight ratio of the activating agent and the introduction of N from urea increased the intrinsic defects, whereas, with rising temperature, the *I*_D_/*I*_G_ ratio increased because of an increase in the concentration of MgCl_2_.^124^

Fig. 4(d and e) show the Raman spectra of AC from *Acacia auriculiformis* tree bark with the expected D and G bands. On deconvolution, the D1 peak can be seen originating from the edge of the carbon atom and the edge planes of the graphitic material, which are orthogonal to the single carbon sheets. The D2 peak (attributed to lattice vibrations), D3 peak (arising from amorphous carbon at the interstitial sites), and D4 peak (lattice vibrations from sp^2^–sp^3^) are also shown.

Table 2 provides a more comprehensive summary of similar findings from Raman analyses, highlighting the intricate levels of control possible in BDAC materials.

**Table 2:** Summary of studies on BDAC graphitization degree based on Raman analysis

Source	Activating agent	Temperature (°C)	*I*_D_/*I*_G_ ratio	Degree	Graphitization	Reference
Garlic sprouts	KOH	800	1.05	—	Highest graphitization	46
Willow catkins	KOH	600	0.79	sp^2^	Highest graphitization	111
Bean dregs	KOH	700	1.19	—	Disordered carbon	126
Tobacco waste	KOH	800	1.10	—	Amorphous carbon	127
Loofah sponge	KOH	800	1.00	—	Disorder and defects	128
Palmyra palm flower	KOH	900	0.89	sp^2^	Disorder and defects	129
Banana peel	Hydrothermal	200	0.84	—	Highly amorphous	130
Onion husk	K_2_CO_3_	800	1.01	—	Disorder and defects	131
Java plum	CO_2_	700	0.99	sp^2^, sp^3^	Amorphous carbon	132
Poovan Kottai	CO_2_	700	0.99	sp^2^	Ordered graphitic structure with defective sites	133
Coconut shell	KOH, ammonium persulfate	700	0.79	—	Highest graphitization	134

From Table 2, it is worth noting that, for SCs, materials having *I*_D_/*I*_G_ ratios between 0.5 and 1.5 are often thought to be promising because conductivity losses and defect concentrations are well-balanced in this range. Values less than 0.5 denote an inadequate number of active sites, whereas values greater than 1.5 indicate high defect concentrations but with limited conductivity.

### 3.5 SEM

SEM reveals the surface morphology of BDAC, providing key insights into the presence and distribution of pores and their size, shape and connectivity. The size and shape of pores determine the adsorption kinetics and mass transfer properties, which allows the indirect assessment of the impact of BDAC preparation conditions, such as different activating agents, the carbonization/activation temperature, and the mass ratio of the activating agent to raw material, on pore development.

**Fig. 5:**
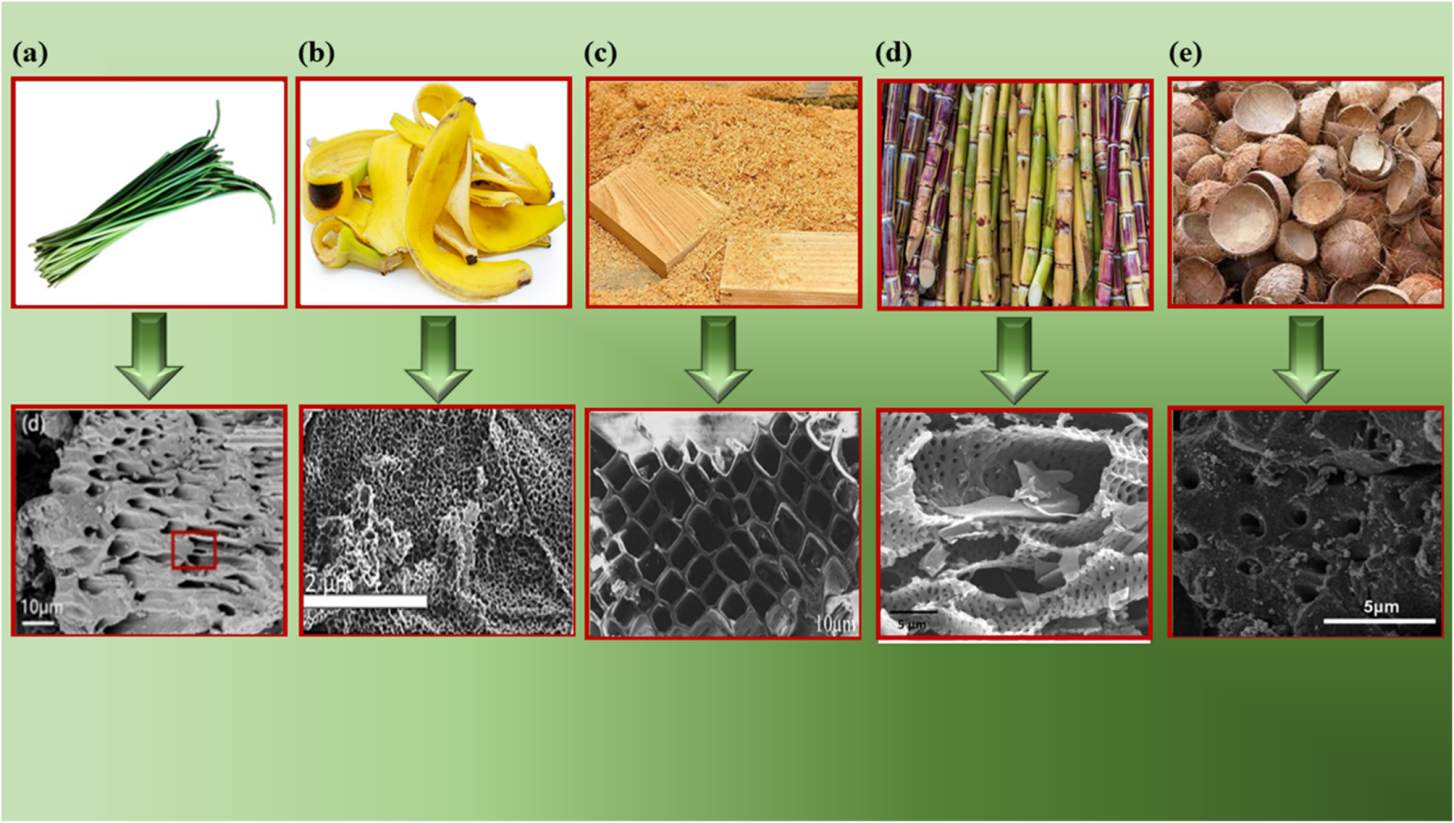
SEM images of different BDAC materials derived from: (a) onion husk (reprinted from ref. 46 under the terms of the Creative Commons CC BY license), (b) banana peel (reprinted with permission from ref. 72 copyright (2020), Elsevier), (c) saw dust (reprinted with permission from ref. 135 copyright (2018), Elsevier), (d) sugarcane bagasse (reprinted with permission from ref. 136 copyright (2018), Elsevier) and (e) coconut shells (reprinted with permission from ref. 137 copyright (2012), the American Chemical Society).

Fig. 5a shows the SEM image of an activated sample derived from garlic sprouts after acid pickling. The material displays a hierarchical pore structure that resembles the vascular bundle and remains intact even after activation and acid pickling. Such hierarchical porous structures can effectively boost the electrochemical performance of the resultant electrodes.^46^

Fig. 5b shows a honeycomb structure of an activated sample derived from the plant cell walls in sawdust.^72^Fig. 5c displays a uniform 3D porous structure derived from banana peels activated at 900 °C, with mesopores and micropores.^133^Fig. 5d represents a microporous carbon structure with folds and pits derived from sugarcane bagasse. These pores were created using an activation temperature of 600 °C, which leads to the specific volatilization of organic components, along with the action of the activating agent (H_3_PO_4_) during impregnation.^136^Fig. 5e illustrates activated carbon derived from coconut shells with a non-uniform and rough surface.^137^

### 3.6 Transmission electron microscopy/high-resolution transmission electron microscopy (TEM/HRTEM)

TEM enables visualization of the internal structures, such as occupancy of the graphitic domains, dislocations, and other disorders.

**Fig. 6:**
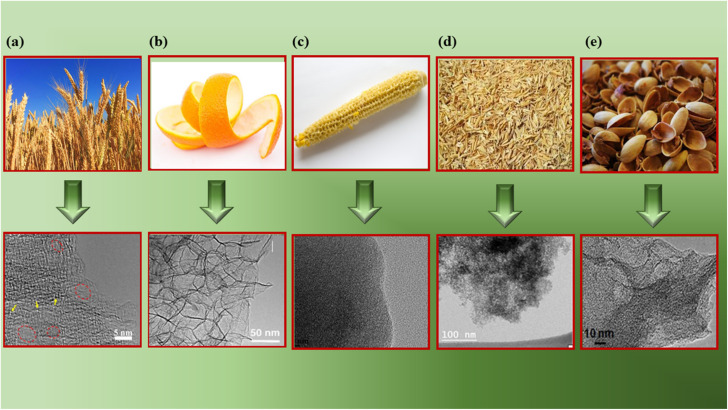
TEM/HRTEM images of various BDAC materials derived from (a) wheat straw (reprinted with permission from ref. 138 copyright (2021), the American Chemical Society), (b) orange peel (reprinted with permission from ref. 139 under a Creative Commons Attribution CC BY 3.0 licence), (c) corn cobs (reprinted with permission from ref. 140 under a Creative Commons Attribution CC BY 3.0 licence), (d) rice husk (reprinted with permission from ref. 141 under a Creative Commons Attribution-Non-commercial 3.0 Unported Licence), and (e) pistachio shells (reprinted with permission from ref. 142 under a Creative Commons Attribution-Non-commercial-No Derivs 4.0 International License).

Fig. 6a demonstrates the activated carbon nanosheets derived from wheat straw; the arrows indicate micropores, and the circles indicate mesopores measuring 3–5 nm.^138^Fig. 6b shows a 3D interconnected network structure of activated carbon derived from orange peel, revealing a wrinkled surface at the outer edges along with few-layered graphene-like areas.^139^Fig. 6c represents the TEM of corn-cob-derived activated carbon with many interconnected micropores and macropores,^140^ and Fig. 6d denotes a hierarchical porous structure with disorder present in activated carbon derived from rice husk. The white spots between the disordered carbon layers indicate the existence of micropores and mesopores.^141^Fig. 6e is an HRTEM image of activated carbon prepared from pistachio nutshells, showing a highly interconnected pore structure with pore sizes in the 0.5–1 nm range.^142^ Hence TEM can help de-convolute and corroborate SEM findings, as well as aid in determining the consistent and uniform internal structure of BDAC.

### 3.7 The Brunauer–Emmett–Teller (BET) technique

BET is crucial for understanding properties, such as surface area, pore size distribution, type of pores, pore volume, and pore diameter.

Of the different types of adsorption isotherms, type I is the Langmuir isotherm, which denotes that adsorption occurs by filling a single monolayer of adsorbate molecules on the adsorbent surface that typically has pores <2 nm.^143^ Type II and III isotherms indicate strong and weak fluid–solid interactions, respectively, for non-porous or macroporous adsorbents. Type-IV and V isotherms exhibit a palpable hysteresis loop due to capillary condensation, indicating that the adsorbent possesses a mixture of mesoporous and microporous regions. Finally, the type-VI adsorption isotherm usually represents a nonporous and highly uniform surface.

According to IUPAC, the hysteresis loop is classified into four parts: H1, H2, H3, and H4. The adsorption and desorption branches in H1 are parallel and almost vertical over a wide range of relative pressures (*P*/*P*_0_), indicating uniform mesopores. H2 has a sharp desorption branch near the lower pressure limit for adsorption–desorption and is triangular, usually exhibited by materials with complex pore structures and non-uniform pore sizes. As for H3, the desorption branch exhibits a steep slope towards the ultimate stages of loop closure, which indicates macropores, whereas H4 possesses parallel and horizontal branches and is exhibited by micro-mesoporous carbon.^144^

**Fig. 7:**
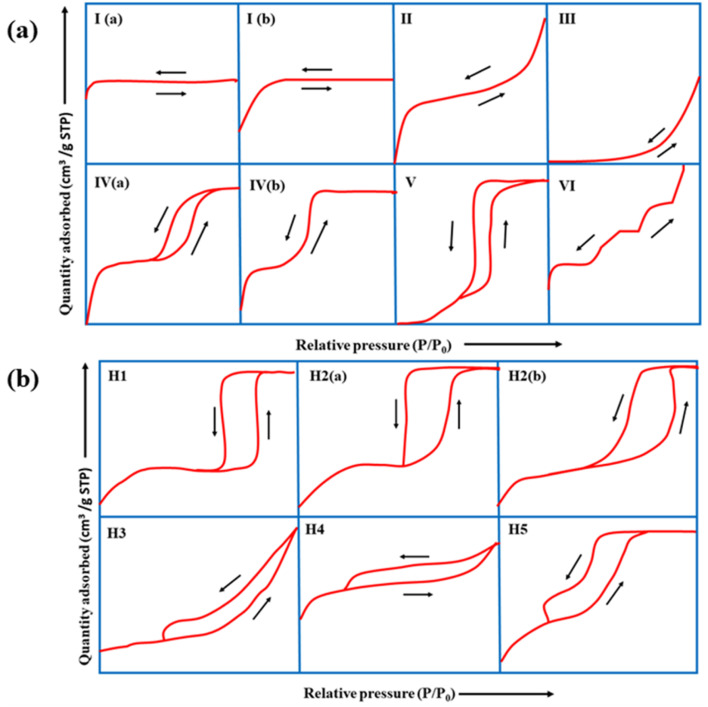
(a) Classification of physisorption isotherms according to IUPAC standards using BET analysis. (b) Types of hysteresis loops as defined by IUPAC standards.

The complex nature of such interpretations establishes that a combination of isotherm types and hysteresis profiles are commonly observed rather than distinct manifestations (as shown in Fig. 7a and b).^49,145^ Among the few BDAC examples investigated, a type II isotherm with an H4 hysteresis loop indicated the presence of a combination of meso and micropores.^42^ Likewise, a type IV with an H3 hysteresis loop demonstrates the dominance of micropores, and a type IV with an H4 loop indicates hierarchical porosity with macro, meso, and micropores^86,146^ manifested by capillary condensation after monolayer–multilayer adsorption. In all BET investigations, the wider the pore size distribution, the higher the adsorbed volume; for instance, a BDAC with a higher number of mesopores correlated with Barrett–Joyner–Halenda (BJH) measurements, which estimated the pore size distributions.^117^

Tables 3 and 4 present an overview of relevant research works, highlighting porosity variations in BDAC, as determined by BET studies.

**Table 3:** Summary of BET investigations that reveal differences in BDAC porosity

N_2_ isotherm curve	Types of isotherm	Inferences
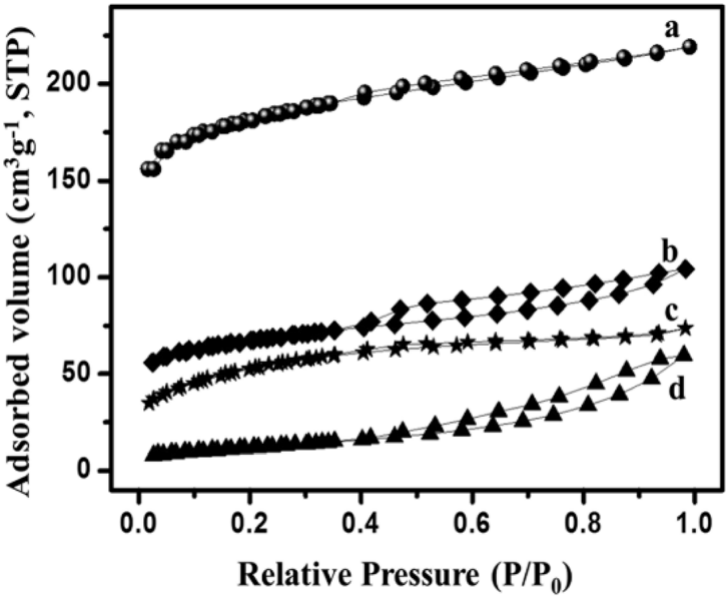	Type IV	(a) Microporosity at *P*/*P*_0_ < 0.45
	Type IV	(b) Macroporosity at *P*/*P*_0_ = 1
	Type IV	(c) High hysteresis at higher relative pressures (>0.4 *P*/*P*_0_)^147^
	Type IV, H3	
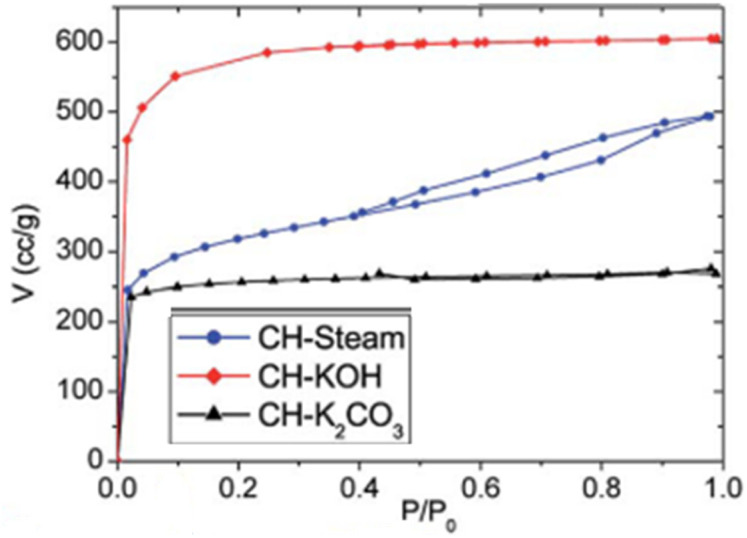	(a) KOH & K_2_CO_3_ – type I	(a) Microporous texture
	(b) Steam – type IV, H3	(b) Micro and mesoporous regions are prevalent^116^
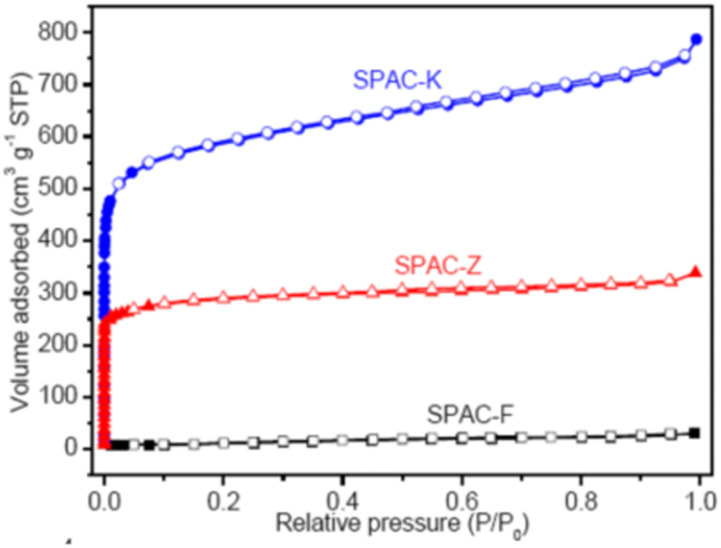	(a) SPAC-F – non-porous material without any adsorption	(a) Non-porous material without any N_2_ adsorption
	(b) SPAC-Z – type I, H2 loop	(b) 0.4 to 0.7 has mesopores
	(c) SPAC-K – hybrid – not conformal to a specific isotherm	(c) 0.1 region reveals micropores. 0.4 to 0.9 with an H2-type hysteresis loop indicates mesopores with different sizes. High relative pressures of 0.9–1.0 indicate macropores^148^
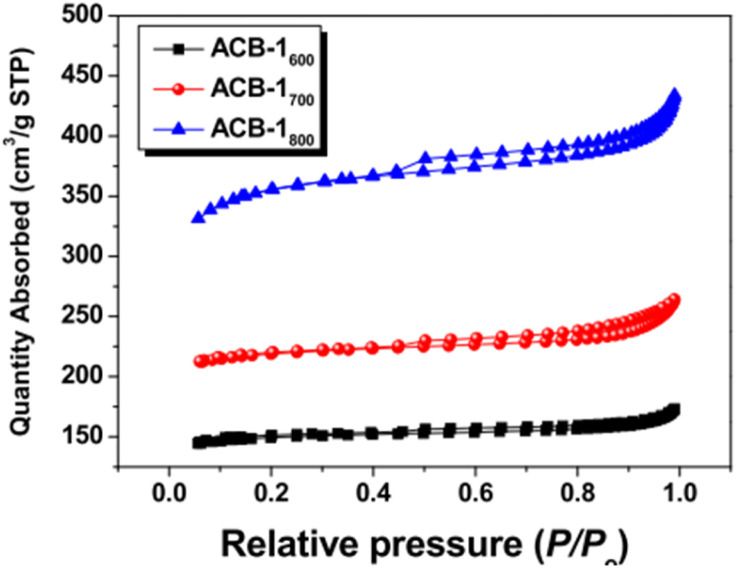	Type II with H3 hysteresis for all samples	Micropores and mesopores without any adsorption even at high relative pressures^125^

**Table 4:** A compilation of different BDAC sources and their manifestations in BET characterization^a^

Source	AA*	Type of isotherm and hysteresis loop	Surface area (m^2^ g^−1^)	Ref.
Dragon fruit	KOH, melamine	Type IV with H4	2994	149
Orange peel	H_3_PO_4_	Type I	2209.7	150
*Thespesia populnea* seeds	KOH	Type IV	1000	151
Pine cone	KOH	Type II with H4	1515	152
Banana fibre	ZnCl_2_	—	1097	
Coconut shell	Steam	Type IV	1559	137
Oil palm kernel shell	Steam	—	730	153

a AA*-activating agent.

**Fig. 8:**
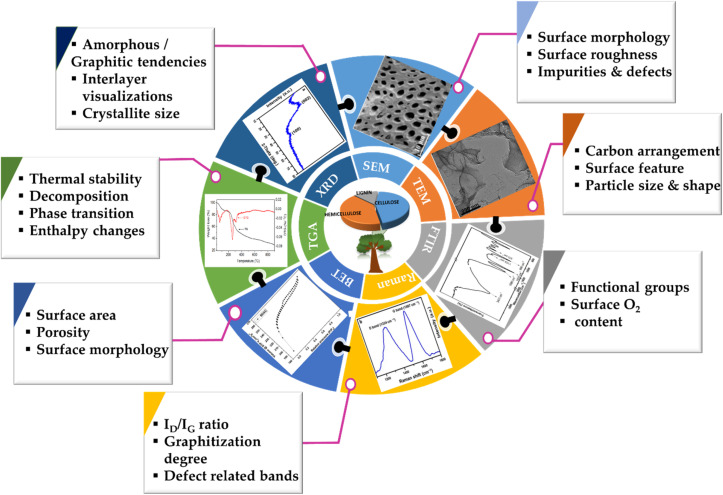
Schematic of multiple techniques used for characterizing BDAC materials.

Through these mainstream characterization tools, the performance capabilities of the myriad BDAC compositions can be universally analysed and benchmarked, aiding the process of continuous property improvement, as displayed in Fig. 8. With these core techniques of material characterization summarised, we delve into the device-level metrics of SCs based on BDACs in the subsequent sections of the review.

## 4 SCs and their characterization

In this part of the review, we discuss the protocols and metrics upon which SCs based on BDAC are constructed and evaluated; this segment is divided broadly into (4.1) generic construction and energy storage mechanisms of SCs and (4.2) the characterization techniques specialized for their evaluation.

### 4.1 Generic principles of SCs

**Fig. 9:**
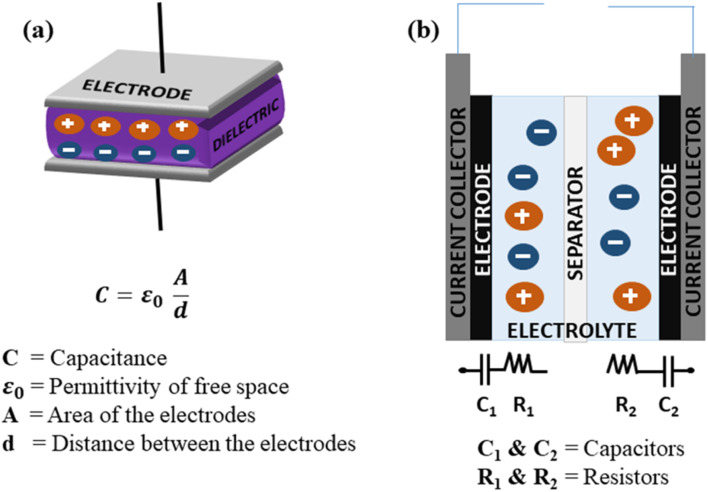
Schematic of the configurations of (a) a dielectric capacitor and (b) an SC component.

SCs generally store energy based on at least two established mechanisms (with the dominant one being dependent on the electrode material used and the type of configuration of the SC): the adsorption/desorption of ions at the electrode/electrolyte interface (termed as the EDLC mechanism) and charge transfer at the bulk near the surface of the electrode (pseudocapacitance-based).^90,154^Fig. 9(a and b) illustrate the fundamental structure of a dielectric capacitor and the corresponding SC.

An electric double layer is formed at the electrode interface *via* the physical adsorption of electrolyte ions.^155,156^ Generally, carbon-based materials like activated carbon, carbon aerogels, carbon nanotubes (CNT), carbide-derived carbons (CDC), and graphene show largely EDLC-based charge storage mechanisms, with little pseudocapacitive behaviour at the surface due to the presence of some O_2_-containing functional groups. However, their pseudocapacitive contribution is less compared with metal oxides or conducting polymers. Carbon materials also have a lower density of active sites and lack multiple oxidation states, which hinder surface redox reactions within the typically limited potential windows that can enhance pseudocapacitive behaviour. EDLC-based SCs exhibit high *P*_d_ and excellent cyclic stability but limited *E*_d_.^157^

Pseudocapacitance-based storage is dominant in SCs made of metal oxides, conducting polymers, transition metal nitrides, transition metal sulfides, perovskites, *etc.*^158,159^ Typically, this behaviour is characterized by redox reactions or reversible faradaic processes that involve electron transfer reactions at the electrode–electrolyte interface.^160,161^ Unlike battery-type materials, this process does not involve any bulk phase changes during charging/discharging.^39^ Since metal oxides have many oxidation states, which can be readily accessed to participate in redox reactions even within the limited potential windows, they predominantly exhibit this behaviour in comparison to the EDLC characteristic, resulting in high *E*_d_ and higher *C*_s_ but low *P*_d_.^162,163^

**Fig. 10:**
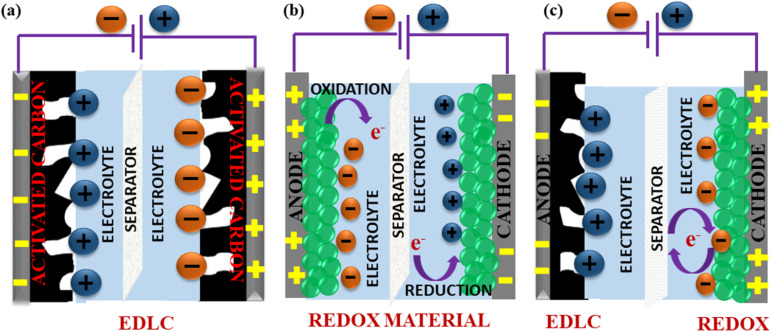
Schematic of the energy storage mechanisms in SCs: (a) EDLC through surface adsorption/desorption, (b) pseudocapacitance based on the surface redox reaction and (c) hybrid SCs with a combination of non-faradaic and faradaic reactions.

Apart from SCs that operate exclusively on either of these mechanisms, hybrid SCs that employ both faradaic (pseudo-capacitance) and non-faradaic (EDLC) based processes to store charge have also been designed. Hence, the advantages include a wide operating voltage window and significant improvement in the *E*_d_ and *P*_d_ of the electrochemical device.^164,165^Fig. 10 shows the mechanisms by which electric charge is stored in an SC.

### 4.2 SC evaluations

**Fig. 11:**
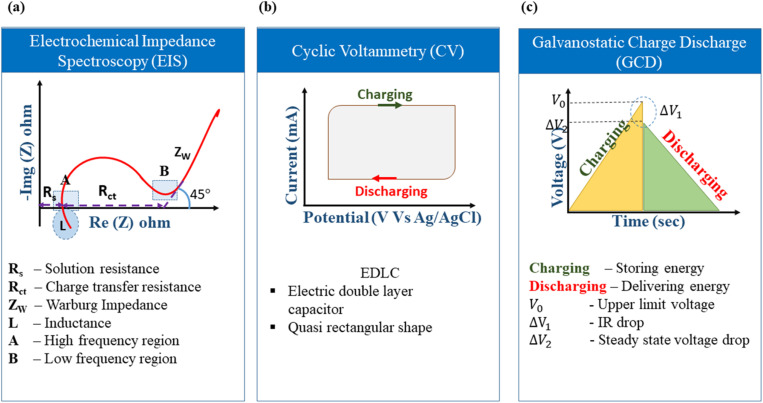
Schematic of electrochemical characterization techniques: (a) EIS (b) CV and (c) GCD.

The elementary understanding of an SC can be obtained using generic metrics like voltage, current, and time *via* electrochemical studies. These studies can be conducted using the following techniques: Cyclic Voltammetry (CV), galvanostatic charge–discharge (GCD), and Electrochemical Impedance Spectroscopy (EIS), the characteristics profiles of which are illustrated in Fig. 11(a–c). These techniques describe multiple parameters, such as operating potential, *C*_s_, ESR, *P*_d_, *E*_d_, capacitance retention, coulombic efficiency, and rate capability. Herein, we briefly explain the mechanisms and principles based on which these characterization techniques allow the calculation of said parameters.

#### 4.2.1 EIS

**Fig. 12:**
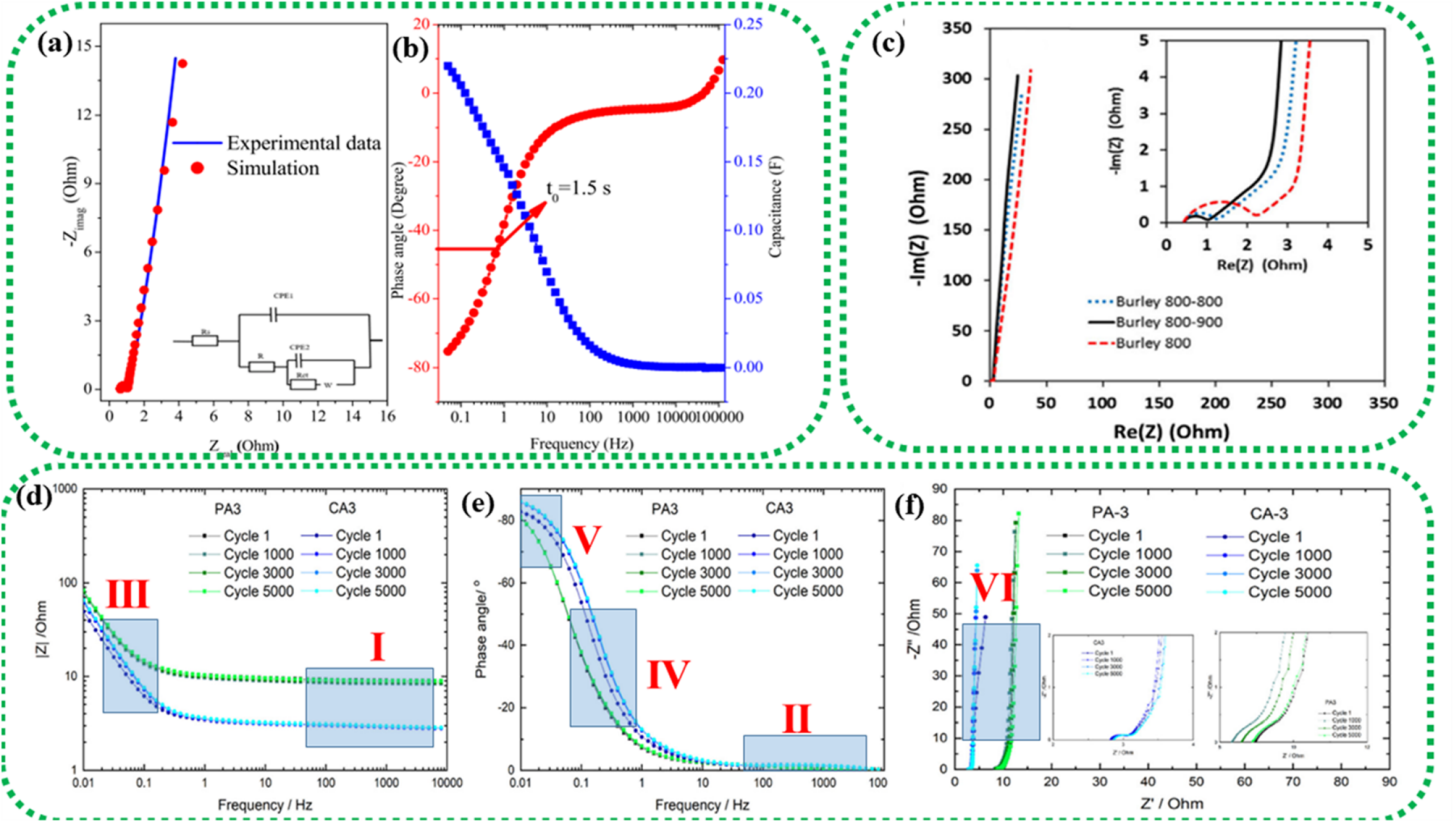
EIS study of an asymmetric device composed of a graphene oxide–polyaniline nanocomposite and AC. (a) Nyquist plot (inset: equivalent circuit model); (b) Bode plot. Reprinted from ref. 172 under the terms and conditions of the Creative Commons Attribution (CC BY 4.0) license. (c) Nyquist plot of two-electrode cells prepared using Burley tobacco-derived AC with different post-treatment temperatures. Reprinted with permission from ref. 173, copyright (2015), Elsevier. EIS characterization of physically (PA) and chemically (CA) activated carbon after 1, 1000, 3000, and 5000 cycles: (d) frequency *vs.* modulus of impedance graph, (e) Bode plot and (f) Nyquist plot. Reprinted with permission from ref. 174, copyright (2021), Elsevier.

EIS is performed galvanostatically by applying an AC potential to the cell and measuring the current passing across the cell due to the double-layer capacitance. It is aimed at quantifying the ESR, charge transfer resistance (*R*_ct_), and phase angle (*φ*). Parameters, such as solution resistance or ohmic resistance (*R*_s_), *R*_ct_, and Warburg impedance (*Z*_w_), are extracted from the EIS study and mapped as the Nyquist plot.^166^ The left portion and right portion in the *X*-axis of the plot correspond to the higher and lower frequency regions, respectively. Typically, at high frequencies, an EDLC-based SC material behaves like a resistor, while at low frequencies, it acts like a capacitor.^167^ The intersection of the semicircle with the Re (*Z*) axis gives the *R*_s_ value arising from the bulk electrolyte, which is also termed ESR.^168^ High ionic resistance of the electrolyte, high intrinsic resistance of the active materials, and poor contact between the current collector and active materials are indicated by a high ESR value.^169^ Resistance dominance is indicated by a broad semicircle, whereas capacitive dominance is denoted by a smaller semicircle. This characteristic behaviour can also be examined by plotting the frequency *vs.* modulus of impedance and *φ*, called a Bode plot. The frequency that coincides with a phase angle of 45° is defined as the knee frequency (*f*_0_) in the Bode plot. At *f*_0_, a transition is observed from higher frequency to lower frequency. Above *f*_0_, the material acts like a resistor and as a capacitor below it; at *f*_0_, both the impedance and capacitive measures of the material are equivalent.^170^ The relaxation time or dielectric relaxation time (*τ*_0_) is defined as the shortest time taken to discharge the device completely. It is also a characteristic figure of merit of SCs as it can dictate the suitability of the SC for high *vs.* low power applications. The experimental relaxation time can be calculated from the plotted graph using the equation *τ*_0_ = 1/*f*_0_, where *f*_0_ is the response frequency of the SC. This relaxation time has an inverse relationship with *f*_0_, that is, a longer *τ*_0_ translates to sluggish kinetics. On the contrary, a shorter *τ*_0_ indicates a higher rate capability. The faradaic charge transfer resistance (*R*_ct_) can be found in the semicircle at the medium-frequency range.^171^Fig. 12a shows the Nyquist plot drawn by fitting the EIS curve to the equivalent circuit model consisting of *R*_s_, *R*_ct_, constant phase element (CPE), and *Z*_w_. The corresponding Bode plot is represented in Fig. 12b, from which the *τ*_0_ can be deduced (1.5 s in this case).^172,175^

The Nyquist plot in Fig. 12c represents the comparison of Burley tobacco-derived AC prepared without (labelled as Burley 800) and with post-annealing treatment (labelled as Burley 800–800 and Burley 800–900) under an N_2_ atmosphere.^173^ It shows a semicircle with an identical ESR value of 0.43 Ω for all ACs. A lower ESR value is typically indicative of a greater concentration of C=C bonds, which can promote electron mobility.^94^ There is also a significant shift in the *R*_ct_ values, from 1.0 Ω (800–900 °C) to 1.25 Ω (800–800 °C) and 2.2 Ω (800 °C). These graphs denoted that the post-treatment helped remove the remaining surface oxygen functionalities, with an accompanying increment in surface area from 1749 m^2^ g^−1^ to 1871 m^2^ g^−1^. The Burley 800–900 °C sample showed a relatively lower *R*_ct_ value of 1.0 Ω, implying optimal conductivity.

The low impedance and phase angle that intercept the *Y* axis close to 0° are shown in Fig. 12(d and e) as regions I and II, respectively.^174^ The semicircle in the mid-frequency region denotes the interfacial contact capacitance related to charge accumulation at the electrode/electrolyte interface and *R*_ct_ arising from both ionic and electronic resistances. The electronic resistance depends on the electrical contact at the interface between the electrode and the current collector, as well as the electrical conductivity of the active electrode material.^176–178^

The ionic resistance is evaluated by the ionic conductivity at the electrode/electrolyte interface. The phase angle tends to change from 0° to −90° with changes in the modulus of the impedance, as shown in Fig. 12d and e in regions III and IV, respectively. The *X*-axis intercept in the lower-frequency region of the Nyquist plot indicates the internal resistance (*R*_int_). In this frequency range, the phase angle tends to reach approximately −85°, suggesting capacitive behaviour, as shown in region V of Fig. 12e (for an ideal capacitor the value should be 90°, the non-compliance is because real materials possess internal resistance, which is usually not zero).^179,180^ This can be identified in the Nyquist plot as a semi-vertical line parallel to the imaginary axis, which is shown as region VI in Fig. 12f. Moreover, the diffusion of solvated ionic species generates additional resistance at the mid-frequency range known as diffusion-controlled resistance *Z*_w_. At higher frequencies, the diffusion of solvated ions is very slow, resulting in a small *Z*_w_. *Z*_w_ can be inferred from the Nyquist plot and Bode plot, as denoted by the tilted line with a 45° slope and the phase angle shift of 45° in Fig. 12f. In certain circumstances, a minor inductance (*L*) behaviour, which may have been brought on by experimental conditions or the internal configuration of the SC, can be present depending also on the cabling and setup,^181^ as shown in Fig. 11a. Thus, Nyquist plots are generally used for probing resistive behavior, while Bode plots give insights into the capacitive nature of the electrochemical system under study.

**Fig. 13:**
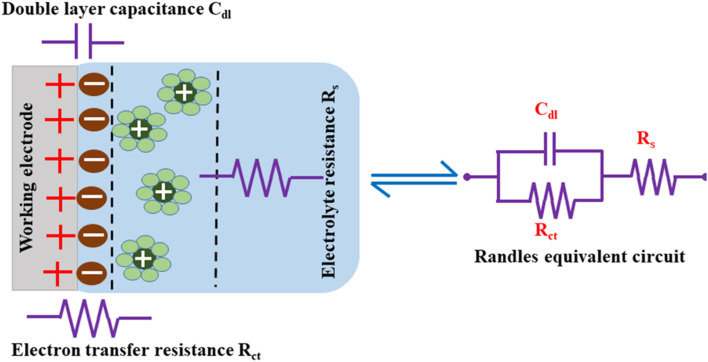
Charge storage *via* EDLC formation and the equivalent circuit.

The measured EIS data are evaluated to understand each component of the electrochemical system by fitting an equivalent circuit model with electrical components, such as capacitors, resistors, and inductors.^182^*R*_s_, *R*_ct_, and *C*_dl_ form the equivalent circuit model, such as that represented in Fig. 13, which also shows the formation of an EDLC layer at the working electrode/electrolyte interface. The resistor (*R*_ct_) and double layer capacitance (*C*_dl_) are connected in parallel, and together they are connected in series to the resistor *R*_s_.

*C*_dl_ indicates the capacitance at the electrode/electrolyte interface. For an ideal capacitor, the electrode should possess a homogenous interface, and the phase angle should be exactly equal to −90°. However, in a real system, the electrode/electrolyte interface is affected by surface roughness, heterogeneity, and uncontrolled instantaneous adsorption/desorption of molecules in the immediate surroundings, leading to non-ideal behaviour. This is represented by a CPE, which is an empirical quantity that correlates the capacitive behaviour with the roughness of the electrode and is given by eqn (15).15
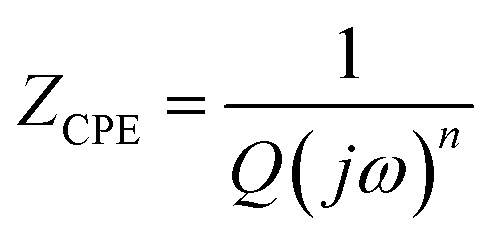
where *Q* represents the proportionality constant, *j* is the imaginary unit that describes the phase angle between current and voltage, and *ω* is the angular frequency. The value of *n* ranges from −1 to 1. If it is 1, the CPE behaves like an ideal capacitor, and as the *n* value changes, the CPE deviates from −90°, representing an increasingly higher resistive or inductive behavior.

Table 5 presents the correlation between CPE values, components, and their respective symbols.

**Table 5:** Representation of CPE values

Value	Elements	Symbol
*n* = 0.5	Warburg	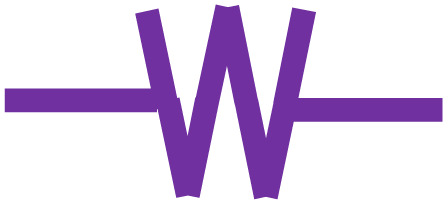
*n* = 1	Capacitor	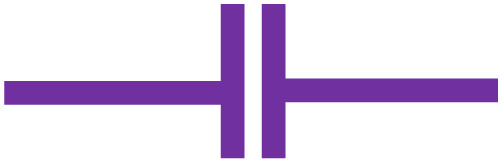
*n* = −1	Inductor	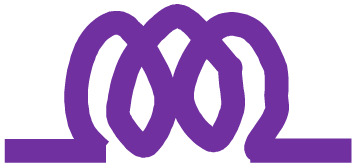
*n* = 0	Resistor	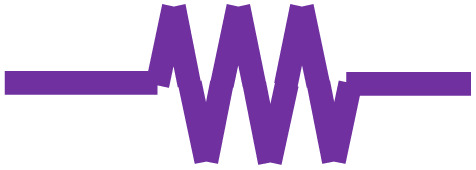

Complex capacitance and complex power analysis are two approaches used in EIS to examine the electrical characteristics of an electrochemical system. They reveal key information regarding the behaviour at the electrode–electrolyte interface. Complex numbers are used to express the impedance 
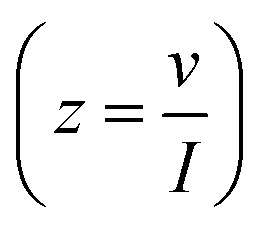
 of the system, accounting for resistance loss and the capacitive behaviour of the system. The phase difference between the current and voltage can then be represented in Farads according to the following eqn (16)–(22),^174,183^

16 *C*(*ω*) = *C*′(*ω*) − *jC*′′(*ω*)

where *C*′(*ω*) and *C*′′(*ω*) are the real and imaginary parts of the complex capacitance *C*(*ω*), respectively, and expressed as,17
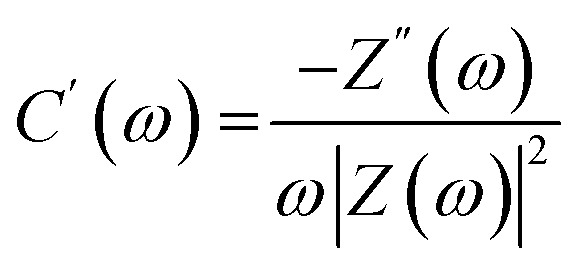
18
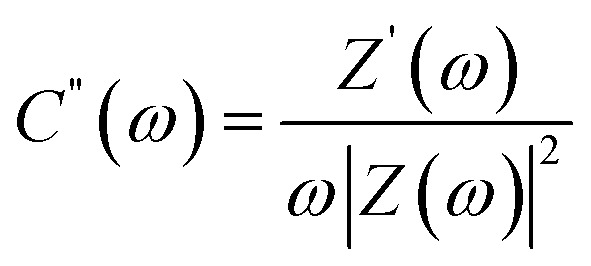
where *Z*′(*ω*) and *Z*′′(*ω*) are the real and imaginary parts of the complex impedance *Z*(*ω*), respectively. *ω* is the angular frequency and is expressed as

19 *ω* = 2π*f*

*C*′(*ω*) represents the capacitance of the electrode material, and *C*′′(*ω*) represents energy dissipation due to irreversible processes, such as self-discharge, leakage currents, degradation of electrode material, and electrolyte breakdown.

The complex power value is given by

20 *S*(*ω*) = *P*(*ω*) + *jQ*(*ω*)

where *P*(*ω*) is the real part of the complex power (termed active power), and *Q*(*ω*) is the imaginary part of the complex power (termed reactive power). These two terms are expressed as

21 *P*(*ω*) = *ωC*′′(*ω*)|Δ*V*_rms_|^2^

22 *Q*(*ω*) = −*ωC*′(*ω*)|Δ*V*_rms_|^2^

where 
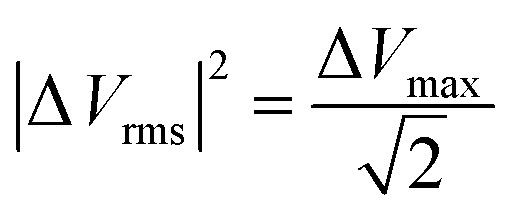
 and Δ*V*_max_ is the maximum voltage.

The SC impedance can be obtained by plotting the real (*C*′(*ω*)) and complex (*C*′′(*ω*)) capacitance values over frequency (*f*).

Moreover, analyzing the distribution of relaxation time (DRT) and the distribution of diffusion time (DDT) extracted from EIS measurements offers crucial insights into the kinetic parameters governing charge transfer, ion diffusion and interfacial parameters within the SC device. These parameters are evaluated within the time domain.^184^ A comprehensive understanding of the fundamental concepts of DRT and DDT can enable researchers to effectively evaluate the performance of SCs.^185–188^

#### 4.2.2 CV

CV is a robust electrochemical tool that measures the current that is developed across an electrochemical cell under voltage sweeping at a given scan rate and voltage over a wide range of limits. In CV, as shown in Fig. 11b, the *X*-axis represents the potential (*V*), and the *Y*-axis represents the measured current (*I*), and information about aspects including but not limited to the redox potential of the molecular species, electron transfer kinetics, voltage windows, *C*_s_ and the reversible/irreversible nature of a process can be obtained.^189^ Since carbonaceous materials tend to exhibit only EDLC behaviour, as discussed in previous sections, this review details the specifics of the CV of EDLC-based materials rather than the pseudocapacitance mode of charge storage.

The ideal CV graph of an EDLC material follows a quasi-rectangular shape, as shown in Fig. 11b. It indicates the unrestricted transport of solvated ions in the activated carbon pores due to the formation of the double layer at the electrode/electrolyte interface.^117^

The *C*_s_ of the device (2-electrode system) can be calculated from the CV data using the following eqn (23):^190^23
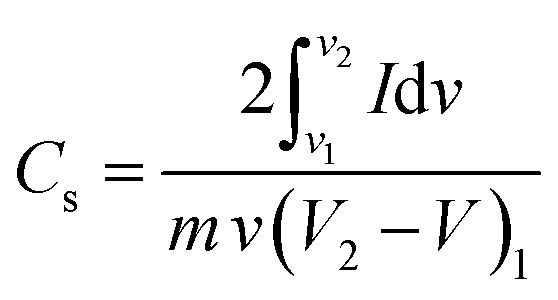
where *C*_s_ is given by F g^−1^, *I* is the current (mA), d*v* is the operating potential window, *m* is the mass of the electrode (mg), *v* is the scan rate (mV s^−1^), *V*_2_ is the upper potential (V) and *V*_1_ is the lower potential (V).

At lower scan rates, the electrolyte ions have sufficient time to access the inner pores of the electrode material and hence offer less ohmic resistance, which enhances the access path for solvated ions in the electrolyte, leading to more charge accumulation on the electrode surface. At a higher scan rate, the CV curve starts deviating from the quasi-rectangular shape because the solvated ions are in contact with the surface for a very short time as a result of the formation of an unstable Helmholtz double layer at the electrode–electrolyte interface, leading to lesser charge accumulation on the surface and a lower capacitance value.^191,192^ Dunn's model is often used to quantify the diffusive and capacitive contributions of an SC system assuming that diffusive contributions are predominant at low scan rates while capacitive contributions become dominant at higher scan rates.^193^

#### 4.2.3 GCD

GCD is a supporting technique for evaluating the capacitance of a device under a constant current. This is also called the constant-current charging/discharging test. GCD records the time taken for charging and discharging an SC device under a constant positive or negative current within a potential limit. In the GCD profile, the *X*-axis represents the time, and the *Y*-axis represents the potential; it is employed to evaluate the capacitance, *E*_d_, and Pd.^194^ The *C*_s_ of a device (2-electrode system) can also be calculated from the discharge curve using eqn (24),24
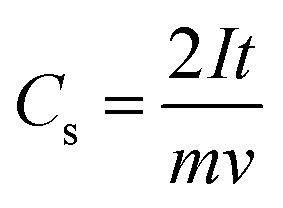
where *C*_s_ is given by F g^−1^, *I* is the applied current (mA), *t* is the discharge time (seconds), *m* is the average mass of both electrodes (mg) and *v* is the voltage (V).

The GCD curves of EDLC-type electrode materials exhibit a symmetric triangular shape during both charging and discharging, as represented in Fig. 10c. This shape is due to a linear voltage change concerning time. *IR* drop, which is defined as the potential difference or voltage drop between the ends of the electrodes during the GCD test, is an important metric that indicates the suitability of a material for SC applications. According to Ohm's Law (Δ*v* = *IR*), an SC experiences a voltage drop when a current (*I*) is passed through it because of the lowering of the internal resistance (*R*). This voltage drop is termed *IR* drop. As the current density increases, the *IR* drop in the GCD curve becomes higher and consequently lowers the discharging time due to faster ionic diffusion.

**Fig. 14:**
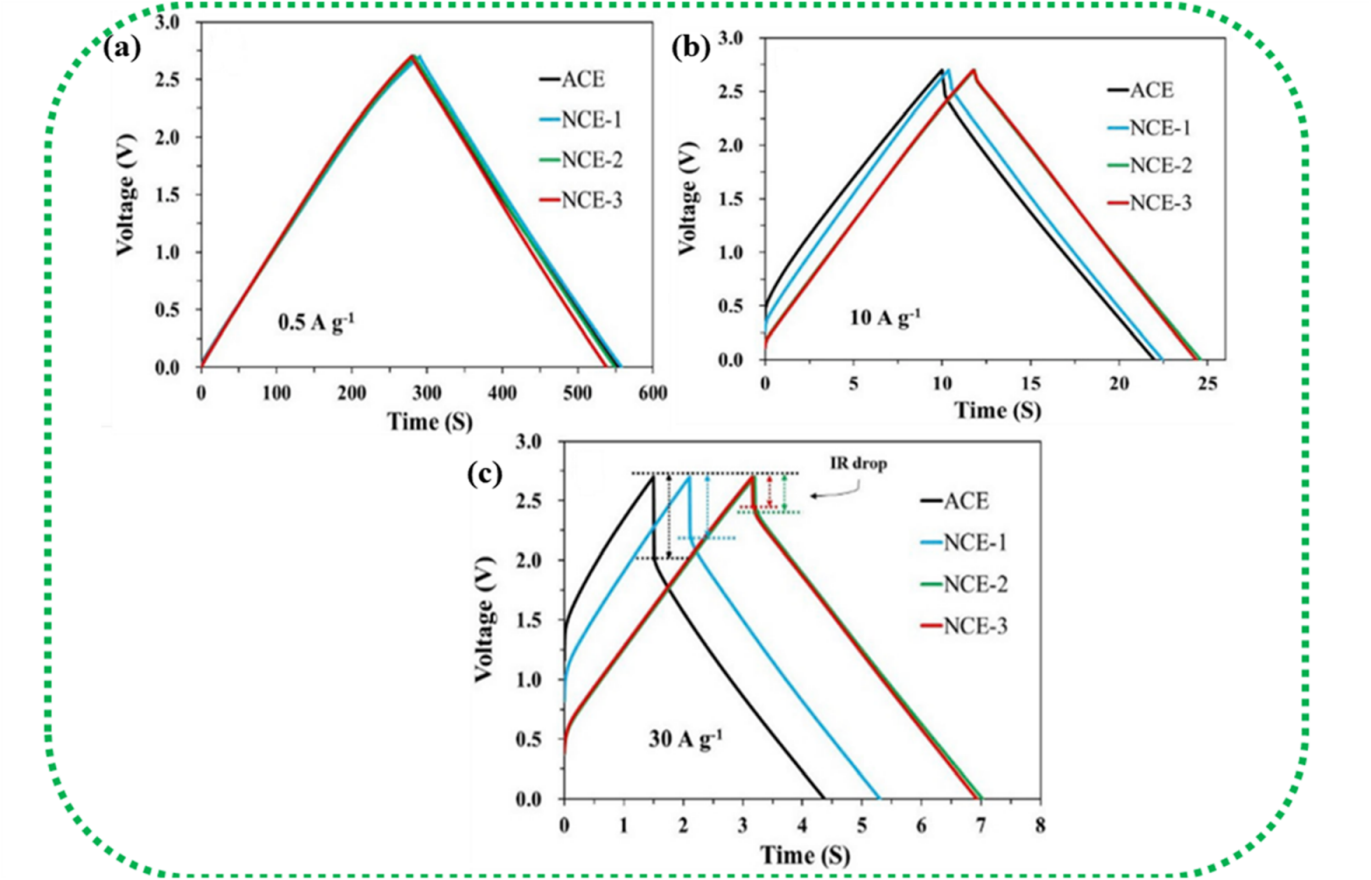
(a–c) Increase in *IR* drop with increasing current density in NCE (AC/CB/CNT/CNF). Reprinted with permission from ref. 195, copyright (2020), Elsevier.

Fig. 14(a–c) show the *IR* drop observed for the various weight ratios of the nanocomposite NCE electrodes at high current densities.^195^ At higher current densities, the electrolyte ions have insufficient time for diffusion into the inner surface of the electrode, resulting in lower capacitance values.^196^*IR* drop is mainly attributed to electrolyte potential drop, contact resistance between the current collector, electrode material, separator, and electrolyte, and the charge–discharge current density. The overall cell performance is deduced by *IR* drop, which is a straight measure of the ESR of the cell. Lower *IR* drops denote lower internal resistance and lead to better coulombic efficiency.^146^

*E*_d_ (W h kg^−1^) and *P*_d_ (W kg^−1^) of an SC can be calculated from the following eqn (25) and (26),^197^25
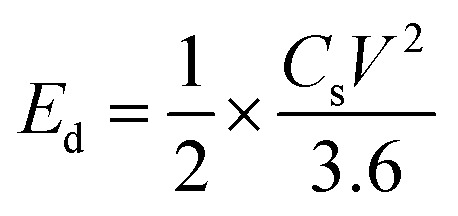
26
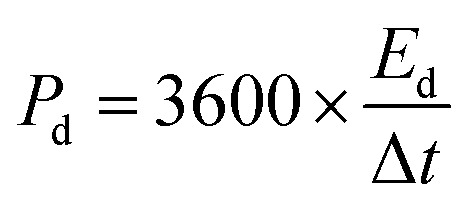


#### 4.2.4 Cyclic stability analyses

Cyclic stability is a crucial factor that assesses the suitability of even the best SCs (as concluded from singular charge–discharge studies) for real applications through multiple and even incomplete charge–discharge cycles.^198,199^ It signifies the capability of an SC configuration to sustain its capacitance over multiple charge–discharge cycles at a specific current density.^172^ The high cyclic stability of SCs denotes a longer lifespan during which they retain their original capacity after many cycles. This test quantitatively reveals the capacitance retention after many cycles and is influenced by the type of electrode material, electrolyte composition, and concentration, as well as test conditions, such as current density, potential window, and temperature. It is always plotted as a function of cycle number to understand the degradation of SCs. A higher value represents good stability and long-term SC performance. The capacitance retention can be deduced from eqn (27):27
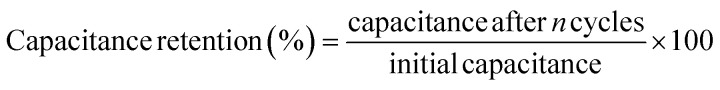


Coulombic efficiency (*η*) is defined as the ratio of time taken to deliver the charge stored during discharge to the time taken for storing the charge and is expressed as a percentage. A value of 100% represents complete discharge without any losses. It is calculated using eqn (28):^200^28
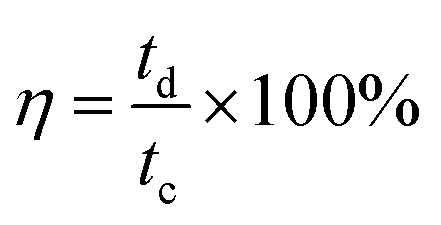
where *t*_d_ is the discharge time, and *t*_c_ is the charging time. Coulombic efficiency is also plotted as a function of the cycle number. A higher *η* indicates lesser energy loss during the charging and discharging processes, suggesting good reversibility of the processes at the electrode/electrolyte interface. Hence, a high *η* value is essential for the commercialization of SCs.

A deeper understanding of conceptualization, methodology, and key factors of the cyclic stability of SCs can be referred from ref. 201.

#### 4.2.5 Voltage holding test (VHT)

The cyclic study is the conventional method for the determination of the stability of an electrode material by subjecting the material to constant-current charge and discharge over several thousands of cycles, after which, ideally, a minimum capacitance loss (and thereby, excellent capacitance retention) should be observed.^202^ During the cyclic tests, the device is charged to the upper threshold voltage and quickly discharged for investigating the constant current phase, and the electrode material is only exposed to the higher voltage for a minimal time.^203^ The VHT or floating test is an alternative to the cyclic test and an effective way of testing the stability limits of the SC device.^204^ In the VHT, the SC is consistently exposed to the upper voltage limit or above the upper voltage limit for a longer duration (typically more than an hour), after which the cell is cycled between the upper and lower voltage limits at a constant current density.^205,206^ During the initial phases of the VHT, typically, the *C*_s_ of the cell exhibits an increase due to the initial stabilizing morphological changes of the porous electrodes (such as expansion and swelling, which consequently augments the available electroactive surface area for ion adsorption within the electrolyte).^152^ This also enhances the wettability of the electrode, which accelerates the diffusion of the hydrated ions between the electrode/electrolyte interfaces and reduces the impedance.^207,208^ After an extended period of floating, the internal resistance of the cell rises while capacitance falls because of the phenomenon of electrolyte starvation, where the electrolyte ions become less available to interact with the inner surfaces of the electrode.^204^ These changes can be easily understood when factors, including but not limited to the diffusion coefficient, surface area, material composition and morphology, porosity, *etc.*, are known. Thus, the SC evaluation protocols complement the core characterization protocols of BDAC very well, as explained in the preceding sections.

#### 4.2.6 Self-discharge test (SDT)

Self-discharge is an implicit attribute of SCs to progressively lose charge over time, even if it's not fixed to any load, as ions migrate from/to the Helmholtz layer until they reach equilibrium, leading to capacitive energy loss, as well as a leakage current.^203^

The presence of ash content, in particular in a BDAC, can have a significant impact on self-discharge, leakage current, and cycling stability.^209^ Ash in BDAC generally comprises mineral components, such as oxides of potassium, sodium, calcium, and silicon, which serve as templates for pore formation. A lower ash content implies a high proportion of fixed carbon content in the raw material.^210^ These minerals can establish a tortuous conductive pathway, which results in the development of a leakage current, directly exacerbating the self-discharge process. One of the key strategies to minimise the ash content is acid soaking; for instance, soaking in HCl before carbonization in commercial potassium humate has been proven effective in reducing the ash content from 20% to 6.3%. As a result, the leakage current reduced (15.3 μA) compared with the material used without acid soaking (25.0 μA) over 5.4 hours of testing.^211^

**Fig. 15:**
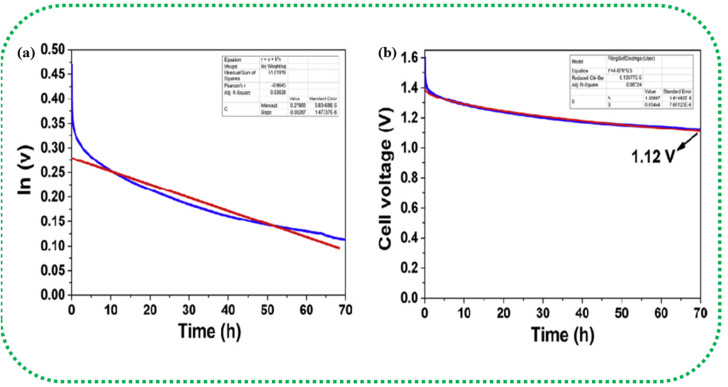
Curve fitting of the self-discharge model. (a) Leakage current over resistance model (b) diffusion control process model. Reprinted with permission from ref. 212, copyright (2017), Elsevier.

The self-discharge mechanism of SCs is explained by two different fitting models, which assume that (a) the internal resistance leads to the development of a leakage current and (b) the self-discharge is predominantly a diffusion-controlled process, as illustrated in Fig. 15(a and b).^213^ The first model correlates the leakage current to the resistance, and the voltage is given by eqn (29),

29 *v* = *v*_0_ exp^−*t*/*RC*^

where *v*_0_ is the initial voltage, and *C* is the equivalent capacitance of the device.

The second model considers self-discharge as a diffusion-controlled process, for this the voltage given by eqn (30),

30 *v* = *v*_0_ − *mt*^½^

where *m* is expressed as eqn (31)^212^31
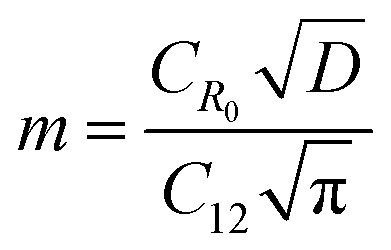
where *C*_*R*_0__ is the excess ionic concentration, *R* is a constant, *D* is the diffusion coefficient of ions in the electrolyte, and *C*_12_ is the series combination of the capacitances at both interfaces of the capacitor per unit area.^214^

#### 4.2.7 Leakage current

Leakage current is associated with self-discharge, during which a low current is dissipated for some time without any external load. Consequently, the device loses energy through heat dissipation as well, which causes complications in thermal management.^215^ The leakage current of the device over a period can be recorded by charging the device to the maximum cell voltage at a particular current density and then maintaining the constant voltage.^216^ A leakage current in the order of μA or nA is preferable for commercial SC applications. Moreover, a higher leakage current can lead to a longer charging time and a higher self-discharge rate.^217^

**Fig. 16:**
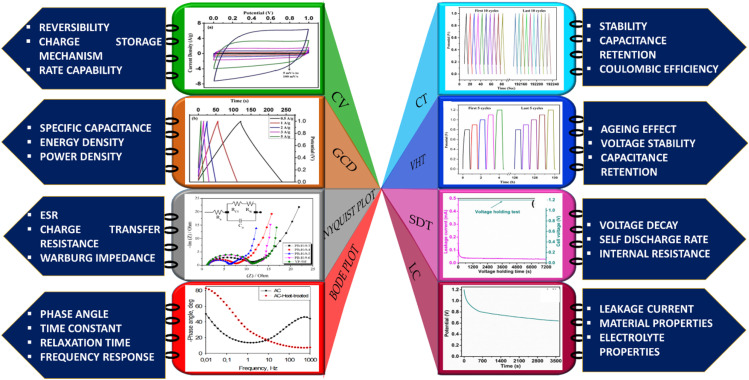
Schematic of the roles of BDAC material in an electrochemical system.

The above sections reveal that apart from CV studies, SC-specific studies can help in specifically judging the stability of a device under high rates of discharge, as well as during inoperative periods, such as storage or no-load conditions. This careful and comprehensive analysis of the performance metrics can be crucial for assessing the suitability of a particular SC configuration before field deployment. Moreover, these tests are highly relevant to BDAC, as BDAC exhibits considerable initial primary changes in metrics, such as surface area, pore hierarchy, surface functionality, interfacial attributes, and homogeneity. Comprehensive BDAC electrochemical characterization and inferences are illustrated in Fig. 16. We next discuss the investigations on peanut shells as a biomass source for BDAC-based SCs. Multiple reviews have summarized the suitability of biomass sources, such as *aloevera*, fermented rice, corn cob residue, oil palm kernel shells, pine cones, shrimp shells, bamboo waste, cotton stalk, sunflower seed shells, waste newspaper, and cauliflower.^218,219^ Considering the geography of our research group *i.e.*, India, we have fittingly chosen peanut shells as the precursor for BDAC-based SCs.

## 5 Peanut shell and its activated carbon for SCs

Globally, peanut shells account for 7.44 million tons of agro-industrial waste annually.^220^ The Food and Agricultural Organization (FAO) reports that India is the second largest producer of peanut products in the world.^221^ Generally, it is used as animal feedstock or dumped in landfills. Peanut shells comprise cellulose, hemicellulose lignin, and ash, which can tailor the surface morphology and electrical conductivity.^222^ Compared to fossil fuels, peanut shells have a low ash content of 1.44% on a dry weight basis.^223^ The elemental analysis of peanut shells (sourced in Argentina and China) using a Carbon Hydrogen Nitrogen Sulphur (CHNS) elemental analyzer has revealed its typical composition, as tabulated in Table 6.^224,225^

**Table 6:** Physicochemical properties of peanut shells

Carbon^a^ (wt%)	42
Nitrogen^a^ (wt%)	2
Hydrogen^a^ (wt%)	7
Oxygen^b^ (wt%)	44
Sulfur^a^ (wt%)	0.08
Moisture^a^ (wt%)	9
Ash^a^ (wt%)	5
Volatile substance	73.80
Fixed carbon	18.62
Cellulose^a^ (g kg^−1^)	41
Hemicellulose^a^ (wt%)	15
Lignin^a^ (wt%)	26

a Air dry basis.

b Calculated by the difference.

Extensive research is currently done in pursuit of innovative electrode materials for the realization of high-performance SC applications. As elucidated in the preceding sections, a multitude of factors are implicated in the customization of material properties. In light of these considerations, a significant number of researchers have considered peanut shells as a suitable raw material for the development of activated carbon. Research on peanut-shell-based BDAC has been categorized based on some common factors considered in published reports, which thereby contribute to the development of sustainable SCs.

### 5.1 Two-step acid pickling

Two-step acid pickling is a promising approach for producing impurity-free activated carbon; this can be done even post-carbonization and activation. Pickling effectively removes impurities, introduces new porosity through *in situ* etching, and improves the accessibility of the carbon matrix for subsequent activation, leading to improved activation efficiency. Effective removal of both metal (and its associated complexes) and other impurities introduced during the activation process is an added benefit, and the formation of new pores has been reported at these positions in the carbon matrix.^226^

### 5.2 Direct activation

High surface area activated carbon (HSAC) has been prepared by single-step direct KOH activation. In addition to this, a binder-free electrode was prepared using this HSAC on nickel foam by a facile hydrothermal method. The electrochemical performance of this electrode was characterized in a three-electrode system, which exhibited a *C*_s_ of 242.84 F g^−1^ in KOH and 272 F g^−1^ in Li_2_SO_4_ electrolyte at a scan rate of 10 mV s^−1^. The higher capacitance in the Li_2_SO_4_ electrolyte is because the direct growth of HSAC on nickel maintains its porous structure; moreover, it favours the insertion of Li^+^ and SO_4_^2−^ ions into the electrode surface during the charging and discharging processes.^227^

Analogously, a single-step method was used to synthesise a composite of PSAC with Co_3_O_4_*via* low-temperature calcination. The PSAC was prepared by carbonization followed by KOH activation. The asymmetric device of this composite displayed a *C*_s_ of 58.2 F g^−1^ at a current density of 1 A g^−1^, with an *E*_d_ of 12.7 W h kg^−1^ and *P*_d_ of 8964.7 W kg^−1^.^228^

### 5.3 Microwave-assisted PSAC

Mesoporous carbons (MCs) have been synthesized from peanut shells *via* single-step microwave-assisted ZnCl_2_ activation. The mass ratio of ZnCl_2_/peanut shell ranged from 1 to 5 (1 : 1, 2 : 1, 3 : 1, 4 : 1, and 5 : 1) at 600 W of microwave power. The specific surface area increased with the mass ratio, from 1307 m^2^ g^−1^ to 1552 m^2^ g^−1^, and then fell to 1409 m^2^ g^−1^ due to pore collapse. On the other hand, the yield of the MCs also declined from 38.4% to 32.3% with an increase in the mass ratio from 2 to 5, likely due to the release of more volatile components at higher concentrations of ZnCl_2._ A mass ratio of 1 : 4 showed optimal parameters, with a *C*_s_ of 184 F g^−1^ at 0.05 A g^−1^ after 1000 cycles.^229^ HPAC prepared by hydrothermal treatment followed by ZnCl_2_ activation had a *C*_s_ of 340 F g^−1^ and a 4.7% decay in capacitance after 10 000 cycles at 1 A g^−1^ in an electrode system with 1 M H_2_SO_4_ as the electrolyte.^230^

Similarly, a microwave-assisted heated glycol reduction method was employed to synthesise a composite of ruthenium/mesoporous carbon from peanut shells.^231^

### 5.4 Hydrothermal carbonization

Hydrothermal carbonization combined with KOH activation was utilized to produce a hierarchical porous carbon with a surface area of 2565 m^2^ g^−1^. The symmetrical device in 3 M KOH showed a *C*_s_ of 188 F g^−1^ at a current density of 0.04 A g^−1^. This device was stable for up to 10 000 cycles with a capacitance retention of 89.3%.^232^

Two distinct methods, hydrothermal carbonization and ethanol soaking were utilized to produce microstructure-activated carbon from peanut shells in another study. Ethanol soaking gave the best result in removing the lignin and hemicellulose components without disturbing the crystalline nature of the cellulose microfibrils. The peanut shell powder was soaked in ethanol for one month to facilitate this removal, which also extended the mesopore range and increased the total pore volume by 250% compared with the material prepared using an alternative hydrothermal process. It revealed higher values, such as a *C*_s_ value of 189 F g^−1^, *E*_d_ of 26 W h kg^−1^, and *P*_d_ of 57 kW kg^−1^, compared with that prepared by hydrothermal carbonization, which showed a *C*_s_ value of 109 F g^−1^, an *E*_d_ of 14.9 W h kg^−1^ and *P*_d_ of 32 kW kg^−1^, which can be attributed to this soaking process.^233^

### 5.5 Activating agents

3D-activated porous carbon materials can be synthesized using different activating agents, such as NaOH, KOH, KCl, and K_2_CO_3_. KCl acts as a template for pore formation in char. Molten KCl replaces the position of volatile substances that are released during the activation process, and KCl is reduced to K, which further intercalates into the carbon network. Consequently, microporous carbon is generated after the elimination of the residual template by acid washing. K_2_CO_3_ has a strong etching effect on the carbon frame, as represented by eqn (32) and (33), because the K atom can readily dissociate during the initial stages of activation and lead to pore formation. CO_3_^2−^ leads to the formation of mesopores as well. The activation mechanism of NaOH is similar to that of KOH, as described in eqn (34)–(36). Nevertheless, the etching effect of NaOH is rather weak because of its lower alkalinity.

32 K_2_CO_3_ + C → K + CO

33 K_2_CO_3_ + C → KO_2_/K_2_O/K_2_O_2_ + CO

34 6NaOH + 2C → 2Na + 2Na_2_CO_3_ + 3H_2_

35 C + H_2_O → CO/CO_2_ + H_2_

36 CO_2_ + C → 2CO

Among the agents listed, BDAC prepared using KOH has shown a high specific surface area of 2936.8 m^2^ g^−1^ and a high *C*_s_ of 339 F g^−1^ at 1 A g^−1^ in a three-electrode system with *E*_d_ up to 39.1 W h kg^−1^ and a *P*_d_ of 2495.5 W kg^−1^ in the two-electrode system. A capacitance retention of 80% after 10 000 cycles at 5 A g^−1^ was also observed.^225^

The investigation of the influence of different activating agents (K_2_CO_3_, KOH, KHCO_3_) at varying weight ratios on PSAC disclosed that among all the activating agents, KOH-derived carbon with a mass ratio of 1 : 4 showed the highest specific surface area of 2547 m^2^ g^−1^, with the symmetric SC showing a *C*_s_ of 224.3 F g^−1^.^234^

The influence of double activating agents was studied using ZnCl_2_ and FeCl_3_ and peanut shells as the raw material. In this work, a single-step activation method was adopted to obtain a ZnCl_2_-activated carbon (ZN-AC), FeCl_3_-activated carbon (FE/AC), and ZnCl_2_/FeCl_3_-activated carbon (ZN-FE-AC). Among all the samples ZN-AC-*x* (*x* = 2 : 1 weight ratio of peanut shell and activating agent) achieved the highest high *C*_s_ value of 239.88 F g^−1^ because it had more ordered carbon and higher O–H and carboxyl group concentrations than the other samples. Though FE/ZN-AC-2 had a high surface area, it showed poor performance. This may be due to the presence of excessive micropores, which will not permit the electrolyte ions to pass through.^235^

The combined influence of ZnCl_2_ and CoCl_2_ as activating agents was examined to prepare an N_2_/O_2_ enriched hierarchical porous carbon (NOHPPC) derived from peanut shells. ZnCl_2_ initiated a dehydrating process during the activation stage, retaining carbon in the matrix. The graphitization degree of the carbon matrix was enhanced through catalytic graphitization using cobalt. The NOHPPC with a weight ratio of 1 : 1(ZnCl_2_ : peanut shell powder) showed a higher graphitization degree (*I*_G_/*I*_D_ = 0.17) than materials prepared using other weight ratios (2 : 1, 3 : 1). It exhibited capacitance retention of 90.9% after 10 000 cycles at 10 A g^−1^ in a three-electrode system containing a 6 M KOH electrolyte.^236^

Porous carbon derived from peanut shell waste was prepared by carbonization and followed by KOH activation with a weight ratio of 1 : 4. This material was used as an anode electrode material along with a sulphur-reduced graphene oxide/cobalt oxide composite in a supercapattery.^237^ Activated carbon obtained from peanut shells using the same method described above was also utilized as anode material in an asymmetric SC configuration along with sulphur-reduced graphene oxide/manganese oxide composite.^238^

### 5.6 The importance of the impregnation ratio

NaOH-based BDAC from peanut shells prepared using various impregnation ratios between 1 : 2 and 1 : 3 showed a rise in specific surface area from 584 m^2^ g^−1^ to 826 m^2^ g^−1^ and pore size from 1.8 nm to 2.1 nm at higher impregnation ratios. This is because higher ratios led to more dehydration, due to which a greater number of ions intercalated into the carbon skeleton, accordingly aiding pore expansion. Moreover, the 1 : 3 impregnated BDAC exhibited a *C*_s_ value of 263 F g^−1^ at 10 mV s^−1^ and 290 F g^−1^ at 0.2 A g^−1^ in a three-electrode system and 98% coulombic efficiency after 1000 cycles.^239^

The utilization of carbon cloth as a current collector and BDAC from peanut shells as the electrode material allowed a high areal mass loading of 9–10 mg cm^−2^. Carbon cloth is corroded by any type of electrolyte, electrically conductive at 15 S cm^−2^ with a porous structure, and has a thickness of 200–250 μm and a high packing density of 0.73 g cm^−3^. With electrolytes, such as 6 M KOH and 0.5 M Na_2_SO_4_, cell voltage values of 1.0 V and 1.6 V have been reported, respectively, for which the differences in the gravimetric and volumetric *C*_s_ of KOH and NaOH (due to different hydrated ionic radii of 0.36–0.42 nm and 0.533 nm respectively) are postulated as the reasons.^240^

### 5.7 Heteroatom doping

#### 5.7.1 *In situ* N doping

The impact of diverse N dopant sources, including urea, urea phosphate, melamine, and ammonium dihydrogen phosphate (H_3_NO_4_P), on PSAC was investigated. Among all N dopant sources, H_3_NO_4_P achieved 3.11% N content, a surface area of 602.7 m^2^ g^−1^ and possessed a higher defect site density and a thicker graphitization layer. Moreover, H_3_NO_4_P acts as an activator as well as an N_2_ source, revealing optimal performance metrics: *C*_s_ of 208.3 F g^−1^ at a current density of 1 A g^−1^ in a three-electrode system and 98.8% capacitance retention at 20 A g^−1^ post 5000 cycles. The symmetric device achieved an *E*_d_ of 17.7 W h kg^−1^ and a *P*_d_ of 2467.0 W kg^−1^ in KOH.^241^

N-doped PSAC prepared using melamine as the N source at a weight ratio of 1 : 2 had a hierarchical porous structure with enriched micropores and mesopores in the range of 2 nm to 5 nm and a surface area of 2014.6 m^2^ g^−1^. Before activation, the raw material was chemically reacted with aqueous NH_4_OH to eliminate lignin and promote its swelling, which increased the etching effect of ZnCl_2_ (the activating agent) during activation.^242^

To enhance the specific surface area, a mixture of peanut shell powder, soy protein as the N source, and GO at a weight ratio of 5 : 1.5 was employed and activated at 800 °C with KOH. The resulting material demonstrated a *C*_s_ value of 289.4 F g^−1^ and capacitance retention of 92.8% after 5000 cycles.^243^

#### 5.7.2 *Ex situ* N doping

Doping carbon skeletons with multiple heteroatoms can imprint a diverse array of functional groups, thus enhancing their physiochemical properties compared to single hetero-atom doping. However, uncontrolled multiple heteroatom doping may result in pore blockage, hindering ion migration within the porous structure, which is a typical problem with *in situ* doping protocols and can adversely affect *C*_s_ and increase resistance. Therefore, careful consideration should be given to the choice of *in situ vs. ex situ* doping in addition to the specific heteroatoms used for optimizing the electrochemical characteristics.

*Ex situ* N doping in the carbon structure is an effective way to enhance the electrochemical properties. The effect of the mass ratio of activating agent in *ex situ* N doping has been investigated using PSAC and melamine the N_2_ precursor, which was mixed at different ratios (1 : 0.5, 1 : 1, 1 : 2) with PSAC and further pyrolyzed at 850 °C for 1 hour in argon. N doping with a 1 : 0.5 ratio achieved a surface area of 1048 m^2^ g^−1^, higher than that of pristine PSAC. The sample with 1 : 1 and 1 : 2 ratios resulted in less porous structures, as evident from SEM and BET analyses, attributed to the decomposition of melamine, which destroys the pore walls and blocks existing pores within the carbon structure. Analogously, *ex situ* N doping with a ratio of 1 : 1 showed the highest *C*_s_ of 251.2 F g^−1^ at a current density of 1 A g^−1^ in a symmetric device configuration compared with other doped and undoped electrode materials. The stability of the device was examined by VHT, and the *C*_s_ values were obtained for every 10 hours of holding time.^244^

#### 5.7.3 N and S heteroatoms were co-doped in biochar from peanut shells through *ex situ* co-doping

Melamine and thiourea were used as the N and S sources. Co-doping led to the refinement of the pore size distribution and the generation of additional active sites for ion adsorption/desorption. The symmetric device fabricated using this PDAC in 1 M H_2_SO_4_ electrolyte displayed a *C*_s_ value of 80.25 F g^−1^ at a current density of 1 A g^−1^ and *E*_d_ and *P*_d_ values of 11.15 W h kg^−1^ and 500 W kg^−1^, respectively.^245^

### 5.8 Molten salt

KCl in its molten state at 800 °C during pyrolysis and subsequent post-heat treatment at a higher temperature (1000 °C) was used to introduce defects and develop the porous structure at the initial stage of PSAC fabrication, which resulted in a *C*_s_ value of 355 F g^−1^ at 0.5 A g^−1^ in the 6 M KOH electrolyte.^246^

### 5.9 Bimetallic activation

A single-step bimetallic activation technique was employed by coupling the activation of metal chlorides (FeCl_3_/ZnCl_2_, FeCl_3_/MgCl_2_, and ZnCl_2_/MgCl_2_) in a CO_2_ environment. The peanut shell powder activated with FeCl_3_/MgCl_2_ at 800 °C attained a *C*_s_ of 247.28 F g^−1^ at a current density of 1 A g^−1^ in a 1 M Na_2_SO_4_ electrolyte with capacitance retention of 96.31% after 5000 cycles. Micropores of size 0.7 nm were observed with a relatively high concentration of oxygen-containing functional groups contributing to the pseudocapacitance response and the highest *C*_s_ value among all the tested activating agents.^247^Table 7 summarizes research done on PSAC for SC applications, including preparation methods, physical properties and SC performance.

**Table 7:** Overview of PSAC derived electrode for SC^a^

Method	AA	Morphology	SSA (m^2^ g^−1^)	Electrolyte	System	*C*_s_ (F g^−1^)	*E*_d_ (W h kg^−1^)	*P*_d_ (W kg^−1^)	Cyclic stability	Retention (%)	Ref.
Conventional method^*^/mechanical exfoliation	KOH	Mesoporous carbon/few-layer graphene	2070	1 M H_2_SO_4_	Three electrode	186 @ 0.5 A g^−1^	58.125	37.5	5000 @ 10 A g^−1^	87	221
Microwave-assisted method	ZnCl_2_	Mesoporous carbon	1552	1 M Et_4_NBF_4_/PC	Three electrode	—	19.3	1007	—	—	223
Conventional method	KOH	3D porous carbon	2936.8	6 M KOH	Two electrode	282 @ 1 A g^−1^	39.1	2495.5	10 000 @ 5 A g^−1^	80	225
Conventional method	KOH	Porous carbon	550.38	6 M KOH	Two electrode	390.9 @ 5 A g^−1^	22.2	319.97	10 000 @ 5 A g^−1^	93.8	226
				PVA/KOH		432.7 @ 0.5 A g^−1^	18.2	275.3			
Hydrothermal/direct growth on Ni foam	KOH	Honeycomb like 3 D porous structure	1279	1 M KOH	Three electrode	242.84	18.89	1000	—	—	227
				1 M Li_2_SO_4_		272 @ 10 mV s^−1^	20	1000			
One-step activation/Co_3_O_4_ composite	KOH	Porous carbon	247.7	6 M KOH	Two electrode	58.2 @ 1 A g^−1^	22.7	8964.7	5000 @ 5 A g^−1^	93.1	228
Microwave-assisted method	ZnCl_2_	Mesoporous carbon	1552	6 M KOH	Three electrode	184 @ 0.05 A g^−1^	4.94	740	1000	—	229
Hydrothermal treatment	ZnCl_2_	Hierarchical porous carbon	1549	1 M H_2_SO_4_	Three electrode	333 @ 0.5 A g^−1^	10.8	6250	10 000 @ 1 A g^−1^	95.3	230
Microwave-assisted method	ZnCl_2_	Mesoporous carbon/ruthenium composite	1176	6 M KOH	Three electrode	287	10.5	—	1000	93.3	231
Hydrothermal carbonization	KOH	Hierarchical porous carbon	2562	3 M KOH	Two electrode	188 @ 0.04 A g^−1^	—	—	10 000 @ 2 A g^−1^	89.3	232
Ethanol soaking/activation	ZnCl_2_	Mesoporous carbon	1483	PVdF-HFP/EMITf/Mg (TF_2_)	Two electrode	189 @ 1.25 A g^−1^	26	57	10 000 @ 1.25 A g^−1^	72	233
Conventional method	KOH	Hierarchical porous carbon	2547	2.5 M KNO_3_	Two electrode	224.3 @ 1 A g^−1^	25.2	900	15 000 @ 5 A g^−1^	83	234
One-step activation	ZnCl_2_ + FeCl_3_	Microporous carbon	1481.59	1 M Na_2_SO_4_	Three electrode	239.88 @ 0.5 A g^−1^	—	—	5000 @ 2 A g^−1^	94.55	235
Conventional method	NaOH	Mesoporous carbon	826	6 M KOH	Three electrode	290 @ 0.2 A g^−1^	—	—	1000 @ 0.2 A g^−1^	90	239
Conventional method	KOH	Microporous carbon	1700	0.5 M Na_2_SO_4_	Two electrode	150.4 @ 0.5 A g^−1^	16.92	451.2	12 000 @ 0.5 A g^−1^	99	240
Hydrothermal carbonization	KOH + H_6_NO_4_P	Mesoporous carbon	602.7	6 M KOH	Three electrode	208.3 @ 1 A g^−1^	18.33	4882.83	5000 @ 2 A g^−1^	98.8	241
Hydrothermal carbonization	ZnCl_2_ + melamine	Hierarchical porous carbon	2014.6	6 M KOH	Two electrode	300.6 @ 0.5 A g^−1^	40.92	990	5000 @ 2 A g^−1^	90.14	242
Conventional method	KOH	Hierarchical porous carbon	1691	1 M H_2_SO_4_	Three electrode	289.4 @ 0.4 A g^−1^	25.72	1280	5000	92.8	243
				PVA/H_2_SO_4_	Two electrode	10.75 @ 10 mV s^−1^			2000	82.9	
Chemical activation/*Ex situ* N2 doping	KOH + melamine	Interconnected porous structure	1442	2.5 M KNO_3_	Two electrode	251.2 @ 1 A g^−1^	35	20	20 000 @ 5 A g^−1^	83.2	244
Conventional method	KOH/melamine + urea	Hierarchical porous carbon	1151.6	1 M H_2_SO_4_	Two electrode	80.25 @ 1 A g^−1^	11.5	5000	5000 @ 5 A g^−1^	98.8	245
One-step bimetallic activation	FeCl_3_ + MgCl_2_	Microporous carbon	1427.28	1 M Na_2_SO_4_	Two electrode	58.83 @ 1 A g^−1^	32.7	588.3	5000 @ 2 A g^−1^	96.31	247
Hydrothermal carbonization	H_3_PO_4_	Mesoporous carbon	17.8	1 M KOH	Three electrode	240 @ 1 A g^−1^	4.08	101.3	1200 @ 3 A g^−1^	90	248
Thermal dissolution carbon enrichment	NaOH	Microporous carbon	2746	6 M KOH	Three electrode	228 @ A g^−1^	—	—	—	—	249
Catalytic graphitization/activation/	CoCl_2_/ZnCl_2_	Hierarchical porous carbon	1942	6 M KOH	Two electrode	221 @ 0.5 A g^−1^	11.1	150	10 000 @ 10 A g^−1^	92.1	236
				1 M [BMIm]BF_4_/AN	Two electrode	135 @ 0.5 A g^−1^	42	375	10 000 @ 10 A g^−1^	89	
Conventional method	KOH	Mesoporous carbon	702.31	2 M KOH	Two electrode	98 @ 0.25 A g^−1^	—	—	20 000 @ 5 A g^−1^	97	68 [our group]

a Conventional method*-carbonization and activation; AA*-activating agent; SSA*-specific surface area; *C*_s_*-specific capacitance.

### 5.10 Other applications

Beyond its use in SCs, PSAC has found applications in H_2_ and CO_2_ storage.^250^ PSAC enriched with micropores and an ultra-high specific area was employed in an H_2_ storage system.^251^ Composites of PSAC with iron oxide have been employed in capacitive deionization to remove chromium ions.^252^ Activated carbons derived from the inner and outer portions of peanut shells were utilized as the anode and cathode material, respectively, in a sodium-ion capacitor,^253^ as well as anode materials in lithium-ion and sodium-ion batteries.^254^ N-enriched activated carbon nanosheets from peanut shells have been used to promote the hydrogen evolution reaction.^255^

**Fig. 17:**
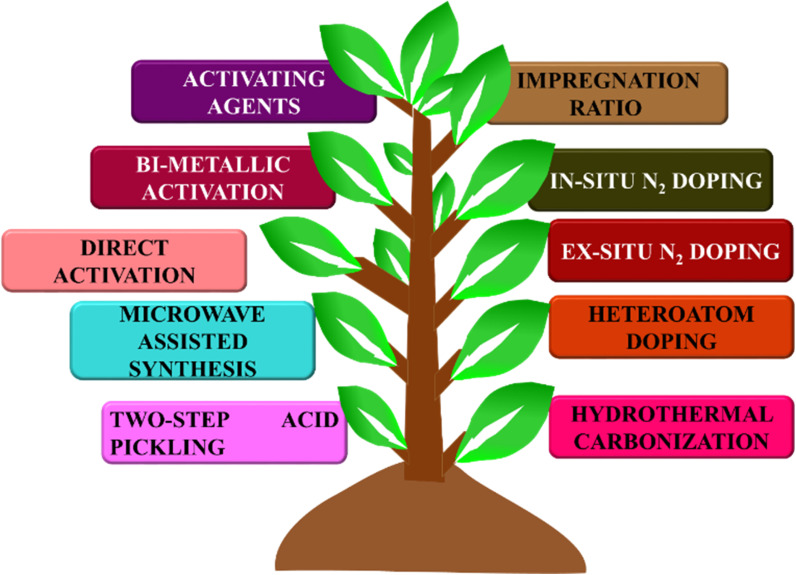
Schematic of critical factors influencing the potential application of PSAC in high-performance SCs.

With the detailing of peanut-derived BDAC and the preceding sections on the parameters governing both BDAC and SCs based on BDAC, we have summarized the extensive research done in these fields by first presenting conclusive observations on various aspects, ranging from the synthesis protocols to contemplations of characterizations and at the device level. We have further highlighted existing challenges and stances on the future outlook of such green sources of SCs as an illustration in Fig. 17.

## 6 Conclusions

The inherent chemical composition, versatile morphology, structural stability, abundance, low cost, and environmental friendliness make BDAC materials promising candidates for SC applications, as detailed in this review. The overview of tailoring the BDAC architecture through activation methods, pre-treatments, activating agents, heteroatom doping, and impregnation time partially drives home this opinion. Unfortunately, there are only a few studies that explore the fundamental factors that influence the design of BDAC materials and the synthesis methodologies. Apart from the overarching conclusions, such as the preference for high carbon content and lower ash content for electrode materials, the intricacies of porosity control and evaluation of its changes over time/cycling, the importance of hierarchical architectures, and specific protocols that can dictate BDAC synthesis *via* an initial component analysis, such as CHNS analyses, have to be addressed for a better standing on the viability of BDAC for commercial applications. Usually, the 3D interconnected hierarchical structure of BDAC materials incorporates a “balanced” distribution of micropores and mesopores, which render them as promising candidates for emerging energy storage devices. This warrants a detailed exploration to understand the optimal hierarchy for specific SC metric targets.

This review elucidates the fundamental characterization techniques employed to analyze diverse biomass sources, including XRD, FTIR, Raman spectroscopy, SEM, TEM, BET, and BET. The assessments of the amorphous nature, structural defect characteristics, and graphitization degrees using XRD, Raman and FTIR analysis of the surface functional groups are detailed to help discern their influence on the wettability and pseudocapacitance behaviour of the electrode materials. SEM and TEM analyses provide insights into the structural morphology and pore distribution, surface area, and pore hierarchy measured by BET analysis, which are critical factors that influence the energy storage capacity of BDAC-based SC devices.

We have also explored the basic principles of SCs, the charge storage mechanisms, and multiple electrochemical characterization techniques, such as CV, GCD, and EIS. The cyclic stability, voltage holding ability, self-discharge, and leakage current (LC) of BDAC electrodes quantified by GCD experiments show interesting aspects, revealing the interactions between the electrodes, electrolytes, and separators during long-term cycling. These results are important for enhancing the cycling stability of BDAC-based electrodes used in SCs.

## 7 Challenges and future outlook

The introduction of innovative technologies is essential to enhancing the production capacity of BDAC and paving the way for commercialisation. This does not necessarily have to be cutting-edge technologies, but more along the lines of significantly better material-level interpretations, especially pre- and post-treatment under real-world operational conditions. Until consistent results are achieved across multiple studies with similar objectives, the randomness associated with BDAC synthesis will always be a hindrance to scaling and realizing precise control in the manufacturing and maintenance of BDAC-based SC devices.

As examples that drive the point that detailed analyses are a necessity, we highlight that self-discharge and leakage current, which are inherent BDAC traits due to the inability to achieve consistent porosity, chemical makeup, and material stability, pose significant challenges to the development of BDAC-based SCs. Implementing strategies, such as solid-state electrolytes and incorporating redox additives into the electrolyte, can effectively mitigate these issues and enhance the overall performance of the device by achieving at least a consistent chemical makeup. Realizing consistent porosity is trickier, as the controlling factors of porosity are countless, ranging from activating agent dosage during biomass pre-treatment to the intricate control of carbonization conditions (temperature, ramp rates, ambients, cool-down rates).

The majority of SC research currently focuses on developing novel electrode materials from different biomass sources and electrolytes, both aimed at enhancing *E*_d_, which is a major bottleneck to SC predominance. Hence to commercialise SCs, it is also rather important to examine factors that contribute to the degradation and ageing of the SCs, including electrode material delamination, electrolyte decomposition, functional group changes, corrosion, gas evolution, phase creation/annihilation, and separator dissolution.

## Data availability

No primary research results, software, or code have been included and no new data were generated or analysed as part of this review.

## Author contributions

T. Manimekala was involved in the conceptualization, analysis, and draft writing of the manuscript. R. Sivasubramanian was involved in the conceptualization, formal analysis, writing, and finalization of the manuscript. Mushtaq Ahmad Dar was involved in the draft formulation, providing critical feedback and preparing the final draft of the manuscript. Gnanaprakash Dharmalingam was involved in funding acquisition, conceptualization, analysis, and manuscript correction.

## Conflicts of interest

There are no conflicts of interest in this manuscript.
